# MZT2B promotes malignant phenotypes in NSCLC cells by enhancing mitochondrial function and COX5B expression

**DOI:** 10.1038/s41419-025-08182-y

**Published:** 2025-11-10

**Authors:** Xinyu Ding, Rongqiang Wei, Chengdong Liu, Zihao Chen, Xiong Qin

**Affiliations:** 1 Department of Thoracic Surgery, Shanghai Pulmonary Hospital, Tongji University School of Medicine, Shanghai, China; 2 Department of Minimally Invasive Thoracic Surgery Center, Second Affiliated Hospital of Naval Medical University, Shanghai, China

**Keywords:** Non-small-cell lung cancer, Oncogenes

## Abstract

Non-small cell lung cancer (NSCLC) remains a leading cause of cancer-related mortality, necessitating the identification of novel therapeutic targets. Here, we identify mitotic spindle organizing protein 2B (MZT2B) as a critical oncogenic driver and potential therapeutic vulnerability in NSCLC. TCGA analysis revealed significant *MZT2B* upregulation in NSCLC tissues, correlating with adverse clinicopathological features and poor prognosis of patients. Single-cell RNA sequencing analysis confirmed predominant *MZT2B* enrichment within malignant epithelial cells, particularly in proliferating carcinoma subsets, across primary tumors and metastatic sites (brain, lymph node, pleural effusions). Functional enrichment analyses highlighted MZT2B’s association with pathways critical for cellular respiration and mitochondrial ATP synthesis, coupled electron transport. Experimental validation in human NSCLC clinical specimens and various cell types further confirmed consistent MZT2B overexpression. Genetic silencing (via shRNA) or CRISPR/Cas9-mediated knockout of MZT2B in various NSCLC cell types significantly impeded cell viability, proliferation, migration, and invasion, inducing G1-S phase cell cycle arrest, and activating the intrinsic apoptotic pathway. Conversely, MZT2B overexpression promoted aggressive malignant phenotypes of NSCLC cells. Further investigation demonstrated MZT2B’s criticality for mitochondrial respiration and overall function, and its silencing or knockout inhibited oxygen consumption rates, ATP production, mitochondrial membrane potential, and cellular redox homeostasis (ROS, GSH/GSSG ratio). Integrated bioinformatic and experimental approaches identified cytochrome c oxidase subunit 5B (COX5B) as a significant downstream effector regulated by MZT2B in NSCLC cells. Restoring COX5B expression or increasing glucose concentration attenuated MZT2B depletion-induced anti-NSCLC cell effects. In vivo studies using subcutaneous xenograft models confirmed that MZT2B knockdown markedly impaired NSCLC tumor growth, reduced proliferation, increased apoptosis, downregulated COX5B expression and diminished mitochondrial function. Collectively, these findings establish MZT2B as a consistently upregulated gene in NSCLC correlating with adverse clinicopathological features and poor prognosis. MZT2B critically regulates mitochondrial function and promotes NSCLC progression, at least partially, through promoting COX5B expression.

## Introduction

Non-small cell lung cancer (NSCLC) constitutes the predominant histological subtype of lung cancer, accounting for approximately 85% of all pulmonary malignancies [1, 2]. NSCLC encompasses two primary subtypes: lung adenocarcinoma (LUAD) and lung squamous cell carcinoma (LUSC), which are distinguished by their cellular origins, genetic profiles, and clinical behaviors [1, 2]. Despite considerable advancements in diagnostic methodologies and therapeutic interventions, NSCLC continues to represent a formidable global health challenge, characterized by its high incidence, aggressive biological behavior, and often unfavorable prognosis, particularly when diagnosed at advanced stages [3–5]. Current standard-of-care treatments encompass surgical resection, radiation therapy, systemic chemotherapy, and targeted molecular therapies [1, 2, 6]. Nevertheless, these approaches frequently encounter significant limitations, including the pervasive development of acquired drug resistance, dose-limiting systemic toxicities, and suboptimal therapeutic efficacy in a substantial proportion of patients [7–9]. These inherent constraints underscore an urgent and critical imperative for the discovery and development of innovative therapeutic strategies [7–9].

The advent of targeted molecular therapies has fundamentally reshaped the treatment paradigm for NSCLC, offering the promise of precise inhibition of specific oncogenic drivers essential for tumor cell proliferation and survival [7, 10, 11]. Agents such as epidermal growth factor receptor (EGFR) tyrosine kinase inhibitors (TKIs) [12] and anaplastic lymphoma kinase (ALK) inhibitors [13], along with immune-checkpoint inhibitors [10], have indeed demonstrated profound clinical benefits, leading to improved progression-free survival and overall response rates in patients harboring specific genetic alterations [7, 10, 11]. However, the applicability of these highly effective therapies remains restricted to a defined subset of NSCLC patients who possess these actionable mutations [7, 10, 11]. Crucially, the inevitable emergence of acquired resistance, often driven by secondary mutations or activation of bypass signaling pathways, consistently diminishes the long-term efficacy of these agents [7, 10, 11]. This persistent challenge necessitates unremitting efforts to identify and rigorously validate novel therapeutic targets that possess the potential to circumvent existing resistance mechanisms and offer broader, more durable clinical utility across diverse patient populations [7, 10, 11].

Mitotic spindle organizing protein 2B (MZT2B) is a mitotic spindle organizing protein localized to the cytosol, microtubule cytoskeleton, and nucleoplasm, specifically within centrosomes and spindle structures [14]. As an integral component of the gamma-tubulin ring complex (γ-TuRC), MZT2B is essential for microtubule nucleation, thereby facilitating the accurate formation and function of the mitotic spindle [14]. This precise organization of microtubules is critical for faithful chromosome segregation during mitosis, which is indispensable for cell proliferation and maintaining genomic stability [14]. Its fundamental biological function lies in its essential role in the organization and regulation of the mitotic spindle, thereby ensuring accurate chromosome segregation during cell division and maintaining genomic stability [14].

Thus, MZT2B, a microprotein component of γ-TuRC, plays a fundamental role in microtubule nucleation and the precise organization of the mitotic spindle [14]. This function is critical for accurate chromosome segregation during cell division, and any dysregulation can lead to genomic instability, a hallmark of cancer. Emerging research links MZT2B to human cancers. High MZT2B expression is significantly associated with gastric cancer, and correlates with positive lymph nodes, distant metastases, and unfavorable patient outcomes [15]. In gastric cancer, its elevated expression is proposed to be activated by the oncogene MYC [15]. MZT2B is also overexpressed in breast cancer, where its knockdown or knockout significantly decreased cell viability, migration, and invasion. Conversely, the tumor suppressor Matrin3 (MATR3) inhibited breast cancer growth by suppressing MZT2B expression (10.1158/1538-7445.SABCS20-PS19-14) [16].

Despite the growing evidence linking MZT2B to cancer, several critical research gaps remain. A deeper understanding of its precise molecular interactions and signaling pathways in human cancer cells is needed. Moreover, detailed functional studies, including gain-of-function and loss-of-function experiments, are essential to rigorously validate MZT2B’s oncogenic roles in other cancer types. Our study is designed to rigorously examine the expression profiles, evaluate the functional consequences of its modulation, and elucidate the underlying molecular mechanisms by which MZT2B influences the malignant phenotype of human NSCLC cells.

## Materials and methods

### Reagents, antibodies and chemicals

All essential cell culture reagents, including basal media, glucose, fetal bovine serum (FBS), and a spectrum of antibiotics, were procured from Hyclone (Logan, UT). Antibodies specific for MZT2A and COX5B (cytochrome *c* oxidase subunit 5 A) were acquired from Abcam (Cambridge, UK); the anti-MZT2B antibody from Thermo-Fisher Scientific (Suzhou, China). All other antibodies were obtained from Cell Signaling Tech (Danvers, MA). A comprehensive array of chemical compounds, such as puromycin, polybrene, and caspase inhibitors, were obtained from Sigma-Aldrich (St. Louis, MO). Fluorescence dye details are reported elsewhere [17, 18].

### Cell and tissue acquisition

Established A549 cells, primary human NSCLC cells (pNSCLC1/-2/-3), and primary lung epithelial cells (pEpi1-2) were all provided by Dr. Sang at Soochow University [17–21]. Prior to experimental use, all cells underwent routine mycoplasma and microbial contaminant screening, short tandem repeat (SRT) profiling for authentication, and rigorous morphological assessment. NSCLC tumor and adjacent normal lung tissues, again provided by Dr. Sang [17–19], were obtained from consenting patients. All human sample protocols received ethical sanction and adhered strictly to Helsinki Declaration principles, fully endorsed by the Ethics Committee of Shanghai Pulmonary Hospital, Tongji University School of Medicine (#BMR-2022-132-SXY).

### Immunohistochemistry (IHC)

Paraffin-embedded xenograft tissue sections underwent preparatory steps: baking, deparaffinization, rehydration, and extensive PBST washes. To mitigate non-specific binding, sections were blocked with 5–6% serum in PBST. Endogenous peroxidase activity was inactivated with hydrogen peroxide. Primary antibody incubation proceeded for 8 h at room temperature, followed by 2 h with biotin-conjugated secondary IgG antibodies. Antigen-antibody complexes were visualized using diaminobenzidine (DAB) chromogen after thorough washing.

### Quantitative real-time polymerase chain reaction (qRT-PCR)

Total RNA was isolated from cell or fresh tissue lysates utilizing TRIzol reagent, then reverse-transcribed into complementary DNA (cDNA). Amplification was conducted according to standardized methodologies [17, 19], with *glyceraldehyde-3-phosphate dehydrogenase* (*GAPDH*) as the internal normalization reference. Quantitative data analysis followed previously validated protocols [17, 19]. Mean values were calculated from five biological replicates. The verified primers specific for *MZT2B*, *MZT2A*, and *COX5B* genes were obtained from Biyuntian (Wuxi, China).

### Western blot

Cellular and tissue lysates underwent separation through SDS-PAGE gels (8%-12% acrylamide), followed by transfer onto polyvinylidene difluoride (PVDF) membranes. Membranes were blocked with 8% non-fat milk in PBST for 55 min, then incubated overnight at 4 °C with primary antibody solutions. After washing via PBST, PVDF membranes were incubated with horseradish peroxidase (HRP)-conjugated secondary antibodies for 70 min at room temperature. Protein bands were visualized via enhanced chemiluminescence (ECL), and densitometric quantification of band intensities was performed using ImageJ software. Mean values were calculated from five biological replicates. Uncropped blot images are included in Fig. S1.

### Single-cell RNA sequencing

As extensively detailed in prior studies [17–19], single-cell RNA sequencing (scRNA-seq) data were processed through Seurat v5.1.0 within the *R* computing environment. This study analyzed two distinct scRNA-seq datasets. The primary dataset, an integrated lung cancer resource available on figshare (10.6084/m9.figshare.c.6222221.v3), offers a comprehensive view of lung cancer transcriptomics [22]. The secondary dataset, GSE131907, was sourced from Kim et al. via the GEO database [23]. Rigorous quality control measures were implemented to remove low-quality cells and technical artifacts. Data normalization was conducted with the SCTransform method to correct technical variations, followed by rPCA (repaired principal component analysis) for batch effect integration. UMAP (uniform manifold approximation and projection) was employed for dimensionality reduction and visualization of cellular heterogeneity. Cell annotations were directly sourced from the original publications of each dataset, maintaining consistency with established classifications.

### Gene silencing and overexpression

For experiments silencing human *MZT2B*, lentiviral constructs were designed and utilized. These included two specific shRNAs for MZT2B (kdMZT2B-sh1/2) with unique sequences for effective knockdown. Constructs encoding *MZT2B* cDNA (hMZT2B[NM_001330282.2]) and COX5B cDNA (hCOX5B[NM_001862.2]) were prepared for overexpression. These lentiviral constructs were co-transfected into HEK-293 cells with essential envelope constructs (Genechem, Shanghai, China) using Lipofectamine 2000 to produce lentiviral particles. These particles, at a multiplicity of infection (MOI) of 12, were used to transduce NSCLC or primary human lung epithelial cells for 40–42 h. Cells were then cultured in medium with polybrene to enhance viral entry, and stable cells were established via puromycin selection over five to seven passages. The efficiency of gene silencing or overexpression was validated at both mRNA and protein levels.

### CRISPR/Cas9-mediated MZT2B knockout (KO)

To achieve precise genetic ablation of *MZT2B*, CRISPR/Cas9-mediated knockout was executed. NSCLC cells were maintained in culture medium with polybrene to aid lentiviral transduction. These cells were transduced with Cas9-expressing lentiviral particles (provided by Dr. Sang [17–19]) to ensure continuous Cas9 nuclease expression. Stable Cas9-expressing cells were selected using puromycin. Two different sgRNAs specifically targeting human MZT2B were used: koMZT2B-sg1 and koMZT2B-sg2. The two were cloned into lenti-CRISPR/Cas9-KO-puro constructs (from Dr. Cao’s group [24, 25]). Lentiviral particles containing the constructs were transduced into stable Cas9-expressing NSCLC cells. Stable knockout cells, labeled “koMZT2B”, were selected using puromycin. Individual stable knockout cells were isolated through single-cell cloning to ensure clonality. Control cells, labeled “sgC” with non-targeting sgRNA, were also from Dr. Sang [17–19].

### Cellular fluorescence staining

For fluorescence staining assays, cells were plated in 24-well plates at 1.6–2.0 × 10^4^ cells per well in 500 µL of basal medium and incubated for a specified duration [17]. After incubation, cells were fixed with 4% paraformaldehyde and thoroughly washed with cold PBS. Specific fluorochromes were applied, followed by additional washes with cold PBS. Visualization was conducted using a Leica epifluorescence microscope, and fluorescence intensity was quantitatively measured with a Hitachi F-7000 spectrophotometer in five random fields at ×100 magnification per treatment. Mean values were calculated from five biological replicates.

### Mitochondrial complex I activity and ATP levels

As reported previously [17, 26, 27], the evaluation of mitochondrial complex I activity was conducted using a Sigma assay kit, employing spectrophotometric techniques to monitor the oxidation of NADH to NAD+, an event catalyzed by complex I. The diminution in absorbance at 435 nm served as the metric for activity. Intracellular and tissue ATP concentrations were ascertained via a colorimetric assay from Sigma, following the manufacturer’s protocol. Each evaluation utilized a 20 µL aliquot of cellular or tissue homogenate comprising 20 µg of total protein. Mean values were calculated from five biological replicates.

### GSH to GSSG ratio

The redox equilibrium, specifically the GSH/GSSG ratio, was quantified using a kit from Thermo-Fisher Scientific (Suzhou, China). Lysates were incubated with DTNB, glutathione reductase, and NADPH, with absorbance measured at 435 nm over a temporal span of 4–5 min using a spectrophotometer. A standard curve derived from bona fide GSH and GSSG standards facilitated precise quantification of concentrations within the lysates. Each analysis was conducted with a 20 µL homogenate sample containing 20 µg of total protein. Mean values were calculated from five biological replicates.

### Single-strand DNA (ssDNA) ELISA

The 96-well plates were coated overnight the purified anti-ssDNA (Novus Biologicals, Shanghai, China), followed by blocking with 1% BSA in PBST. Lysate samples and controls were incubated for 90 min at ambient temperature, then detected with peroxidase-conjugated anti-human IgG for 45 min. Subsequent washes preceded the addition of TMB (3,3′,5,5′-Tetramethylbenzidine) substrate for chromogenic development, halted with H_2_SO_4_, and absorbance was recorded at 445 nm, referencing 615 nm. Mean values were calculated from five biological replicates.

### Transwell assays

For in vitro migration assays, genetically engineered NSCLC cells were apportioned at 1.3 × 10^4^ cells per well in serum-deprived medium within Transwell chambers. Following a 24-h incubation, cells that had traversed to the basolateral surface were fixed, stained, and visualized. In in vitro invasion assays, chambers were coated with Matrigel (Sigma), with all other parameters remaining constant. The number of migrated/invaded cells was quantified by counting cells in five random fields per well at ×100 magnification. Mean cell numbers were calculated from five biological replicates.

### Oxygen consumption rate (OCR)

OCR was delineated using an Agilent Seahorse XF24 Extracellular Flux Analyzer, adhering to established protocols [28]. Cellular respiration was scrutinized by sequentially administering metabolic modulators: 1 µM oligomycin, followed by 0.5 µM FCCP [carbonyl cyanide-4-(trifluoromethoxy)phenylhydrazone], and concluding with 0.5 µM antimycin A and rotenone. This pharmacological perturbation enabled comprehensive quantification of basal, ATP-linked, maximal, and non-mitochondrial OCR components, normalized to intracellular protein content. Mean values were calculated from five biological replicates.

### Additional cellular assays

A plethora of functional cellular assays were executed as delineated in prior studies [17, 26, 27]. Genetically modified NSCLC or lung epithelial cells were seeded at 3000 cells per well in 96-well plates and incubated for a designated duration. A CCK-8 solution was subsequently added, followed by a 100-min incubation, with absorbance measured at 450 nm via a microplate reader. Mean optical density (OD) were calculated from five biological replicates. Caspase-3 and -9 activities in cell lysates were assayed using a Caspase-3/-9 colorimetric kit (Biyuntian, Wuxi, China), adhering stringently to the manufacturer’s protocol. Mean values were calculated from five biological replicates. Each treatment involved 20 µL of lysate containing 20 µg of total protein. Trypan blue staining with an automatic cell counter determined cell viability post-treatment. The counter’s software automatically identified and counted unstained (viable) and blue-stained (dead) cells, from which the percentage of dead cells was calculated. The mean dead cell ratio was calculated from five biological replicates. FACS assays of cell apoptosis and cell cycle progression were also reported previously [17, 18, 21]. In brief, apoptosis analysis was conducted using the Annexin V-FITC/Propidium Iodide (PI) Apoptosis Detection Kit (Catalog #A13202, Thermo Fisher Scientific) according to the manufacturer’s specifications. Following treatment, cells were washed with cold PBS and resuspended in 1× binding buffer. The cells were then stained with Annexin V-FITC and PI for 15 min at room temperature in the dark. For cell cycle analysis, cells were fixed overnight in cold 70% ethanol at 4 °C. Fixed cells were washed with PBS and resuspended in a staining solution containing 40 µg/mL PI and 100 µg/mL RNase A for 30 min at 37 °C. In both assays, fluorescence was measured on a BD FACSCanto II flow cytometer (BD Biosciences). Data acquisition and analysis were performed using FlowJo software (v10). The percentages of cells in different cell cycle phases (G 1, S, and G 2) or in the stage of apoptosis were quantitatively determined from the resulting histograms, with mean values calculated from five biological replicates.

### Animal studies

Previously established protocols [17, 18] guided the in vivo xenograft studies, which utilized six-week-old nude mice (17.5–18.2 g) with an equal distribution of sexes. Animals were housed at the Animal Facility at Soochow University (Suzhou, China). Subcutaneous injection of 6 × 10^6^ pNSCLC1 cells per mouse into the flanks initiated tumor development. Three weeks after cell injection, tumor dimensions were measured, and volumes were calculated using the aforementioned established formula [17, 18]. All animal experimental protocols received ethical approval from the Institutional Animal Care and Use Committee (IACUC) and the Ethics Committee of Shanghai Pulmonary Hospital, Tongji University School of Medicine (#BMR-2022-055-SXY).

### Statistical analysis

In vitro cellular studies consistently employed a rigorous blinded approach for group allocation and were replicated across five distinct biological repetitions. All data exhibited normal distribution and were presented as mean ± standard deviation (SD). Statistical analyses were performed using SPSS version 26.0 (SPSS Co., Chicago, IL). The unpaired Student’s t-test was utilized for comparisons between two specific groups. For comparisons involving more than two groups, one-way ANOVA was applied, followed by post-hoc Scheffe’ and Tukey tests. The statistical methods used for the bioinformatic analyses of The Cancer Genome Atlas (TCGA) data are also detailed. Statistical significance was defined as a *P*-value < 0.05.

## Results

### TCGA-based analysis shows upregulated *MZT2B* expression correlating with adverse clinicopathological features and poor prognosis in NSCLC

**Fig. 1:**
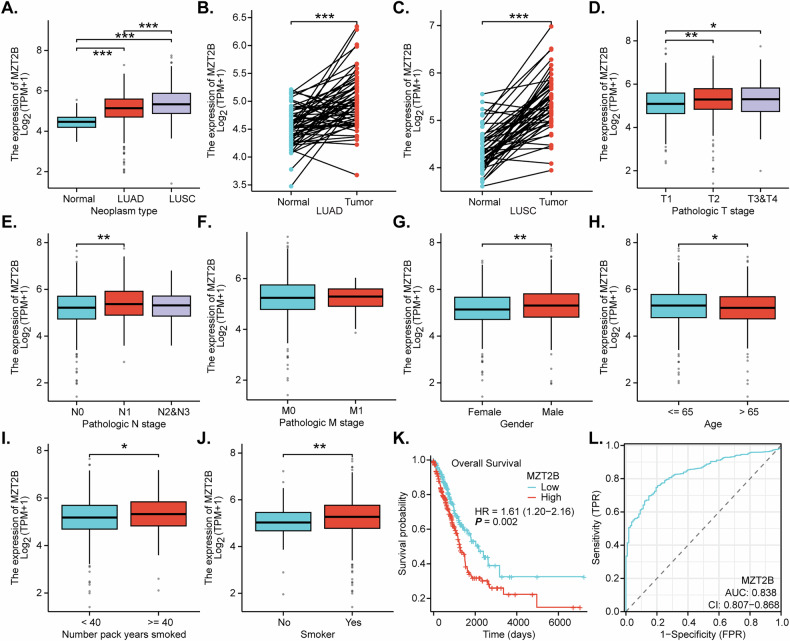
TCGA-based analysis shows upregulated *MZT2B* expression correlating with adverse clinicopathological features and poor prognosis in NSCLC. Transcriptomic analyses of The Cancer Genome Atlas (TCGA) datasets were undertaken to measure the differential expression of *MZT2B* in neoplastic lung tissues, specifically lung adenocarcinoma (LUAD) and lung squamous cell carcinoma (LUSC), relative to their normal counterparts (**A**). Subsequent direct comparisons were carried out on matched tumor-normal tissue pairs to delineate *MZT2B* expression profiles within LUAD (**B**) and LUSC (**C**) tumor specimens juxtaposed with their adjacent normal pulmonary tissues. Concomitant analyses explored the association of *MZT2B* expression with a spectrum of clinicopathological attributes, encompassing pathological T stages (T1 versus T2, T3&T4) (**D**), the presence and extent of lymph node involvement (N0 versus N1, N2&N3) (**E**), metastatic dissemination to distant sites (M0 versus M1) (**F**), patient sex (**G**), age demographics (≤65 versus >65 years) (**H**), cumulative smoking exposure expressed in pack-years (<40 versus ≥40) (**I**), and current/former smoking habit versus never-smoker status (**J**) in NSCLC cohorts. Kaplan–Meier survival methodologies were employed to ascertain the correlative relationship between *MZT2B* expression levels and overall survival (OS) among NSCLC patients (**K**). The prognostic utility of *MZT2B* expression in NSCLC was further evaluated through receiver operating characteristic (ROC) curve analysis (**L**). HR hazard ratio, CI confidence interval, TPM transcripts per million, FPR, false positive rate, TPR true positive rate. The Kruskal–Wallis post hoc Dunn test was used in (**A**, **D**, **E**), the Wilcoxon signed rank test in (**B**, **C**). Mann–Whitney U test in (**F**–**J**), Cox regression analysis in (**K**). Asterisks signify levels of statistical significance: *P < 0.05, **P < 0.01, ***P < 0.001.

We first carried out an analysis of *MZT2B* expression in NSCLC based on The Cancer Genome Atlas (TCGA), showing *MZT2B* expression was notably elevated in NSCLC tissues. Specifically, both LUAD and LUSC demonstrated significantly higher *MZT2B* levels when compared to normal lung tissues (Fig. 1A). This upregulation was particularly evident in paired tumor samples, where *MZT2B* expression was markedly increased compared to adjacent normal tissues in both LUAD (Fig. 1B) and LUSC (Fig. 1C). An analysis of *MZT2B* expression in relation to clinicopathological features revealed several significant associations. Higher pathological T stages (T2, T3&T4) exhibited elevated *MZT2B* expression compared to T1 stages (Fig. 1D). Furthermore, *MZT2B* levels were significantly increased in patients with advanced pathological N1 stages in comparison to those with N0 stage disease (Fig. 1E). However, no significant difference in *MZT2B* expression was observed between M0 and M1 pathological M stages (Fig. 1F). Gender-based analysis indicated that male patients presented with higher *MZT2B* expression than female patients (Fig. 1G). *MZT2B* expression was significantly higher in younger patients (≤65) than that in the aged patients (>65) (Fig. 1H). Regarding smoking history, patients with ≥40 pack-years exhibited significantly higher *MZT2B* expression than those with <40 pack-years (Fig. 1I). Consistently, individuals classified as current or former smokers demonstrated significantly elevated *MZT2B* expression compared to never-smokers (Fig. 1J). The prognostic utility of *MZT2B* in NSCLC was further investigated. Kaplan–Meier survival analysis unequivocally demonstrated that high *MZT2B* expression was significantly correlated with diminished overall survival in NSCLC patients (Fig. 1K, *P* = 0.002, HR = 1.61, 95% CI: 1.20–2.16). Complementing this, receiver operating characteristic (ROC) curve analysis highlighted the robust predictive capability of *MZT2B* expression for NSCLC prognosis, yielding an area under the curve (AUC) of 0.838 (95% CI: 0.807–0.868) (Fig. 1L). These findings establish *MZT2B* as an upregulated gene in NSCLC that significantly correlates with adverse clinicopathological characteristics and serves as a robust indicator of poor patient prognosis.

### Single-cell RNA sequencing analysis of integrated NSCLC data reveals *MZT2B* enrichment in malignant epithelial cells and associated functional pathways

**Fig. 2:**
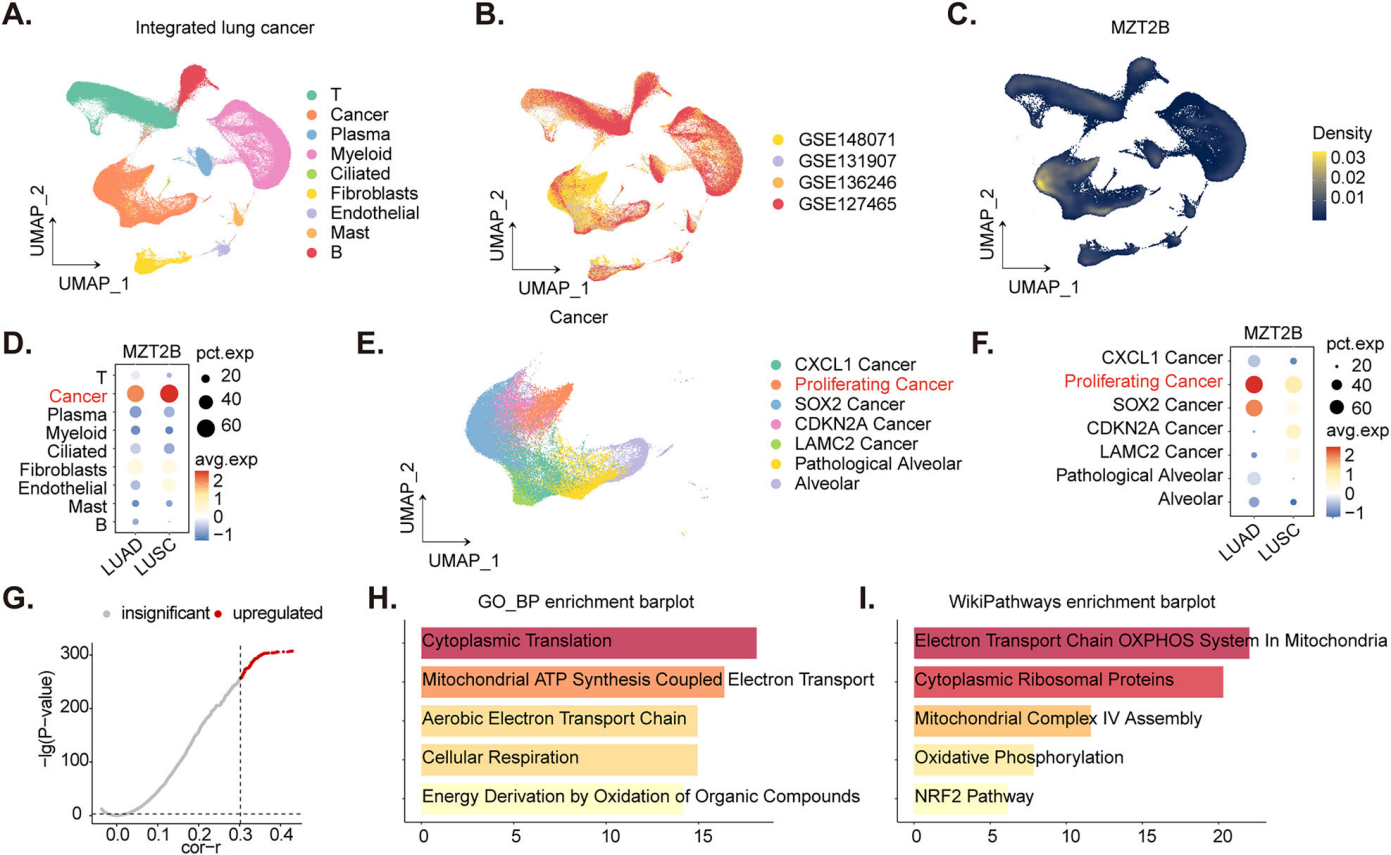
Single-cell RNA sequencing analysis of integrated NSCLC data reveals *MZT2B* enrichment in malignant epithelial cells and associated functional pathways. UMAP (uniform manifold approximation and projection) visualization of integrated single-cell lung cancer data, colored by cell type annotations provided by original authors (**A**). UMAP visualization of integrated single-cell lung cancer data, colored by original data source (**B**). UMAP visualization showing the expression density of *MZT2B* across all integrated cells (**C**). Dot plot illustrating the percentage of cells expressing *MZT2B* and average expression levels across different cell types in both LUAD and LUSC, highlighting predominant *MZT2B* expression in malignant epithelial cells and higher expression in LUSC (**D**). UMAP visualization of re-clustered malignant cell populations from integrated lung cancer data, colored by sub-cluster identity (**E**). Dot plot displaying the percentage of cells expressing *MZT2B* and average expression levels across different malignant cell sub-clusters, demonstrating high *MZT2B* expression in proliferating cancer cell subsets (**F**). Correlation analysis of *MZT2B* with other genes within cancer cell subpopulations, showing the top 100 positively correlated genes (**G**). Bar plot depicting enriched Gene Ontology (GO) biological processes for genes positively correlated with *MZT2B* expression (**H**). Bar plot illustrating enriched WikiPathways for genes positively correlated with *MZT2B* expression (**I**).

Next, we leveraged an integrated single-cell RNA sequencing dataset derived from NSCLC, with cellular annotations meticulously provided by the original authors [22]. Initial dimensionality reduction projections (Fig. 2A) visually elucidated the distinct cellular populations based on the provided annotations, concurrently illustrating the compositional contribution of various integrated data sources (Fig. 2B). An assessment of *MZT2B* expression density (Fig. 2C) unequivocally demonstrated its predominant localization within the malignant cellular compartment. Quantitative analysis further revealed a substantial upregulation of *MZT2B* within these cancer cell aggregates (Fig. 2D), with a particularly increased expression observed in LUSC compared to LUAD (Fig. 2D). To ascertain a finer resolution of *MZT2B* distribution, the malignant cellular population was carefully extracted and subjected to sub-clustering analysis (Fig. 2E, F). This refined segmentation corroborated the elevated *MZT2B* expression within these sub-clusters, exhibiting significant enrichment within proliferating carcinoma cell subsets (Fig. 2E, F).

To elucidate the functional implications underpinning *MZT2B* expression within the tumoral milieu, a comprehensive correlational analysis was carefully executed across the identified cancer cell subpopulations. This investigation identified the top 100 genes exhibiting robust positive correlation with *MZT2B* expression (Fig. 2G). Subsequent multifaceted functional and pathway enrichment analyses were rigorously applied to this gene set. Both Gene Ontology (GO) biological process enrichment (Fig. 2H) and WikiPathways enrichment (Fig. 2I) consistently delineated significant enrichment in processes pivotal to cytoplasmic translation, the biogenesis of cytoplasmic ribosomal proteins, cellular respiration, mitochondrial complex IV assembly, and mitochondrial ATP synthesis coupled electron transport (Fig. 2H, I). Collectively, these findings underscore a pivotal role for *MZT2B* in the orchestration of critical mitochondrial function and metabolic mechanisms within the context of NSCLC carcinogenesis.

### *MZT2B* is consistently overexpressed in malignant epithelial cells across primary and metastatic sites in LUAD

**Fig. 3:**
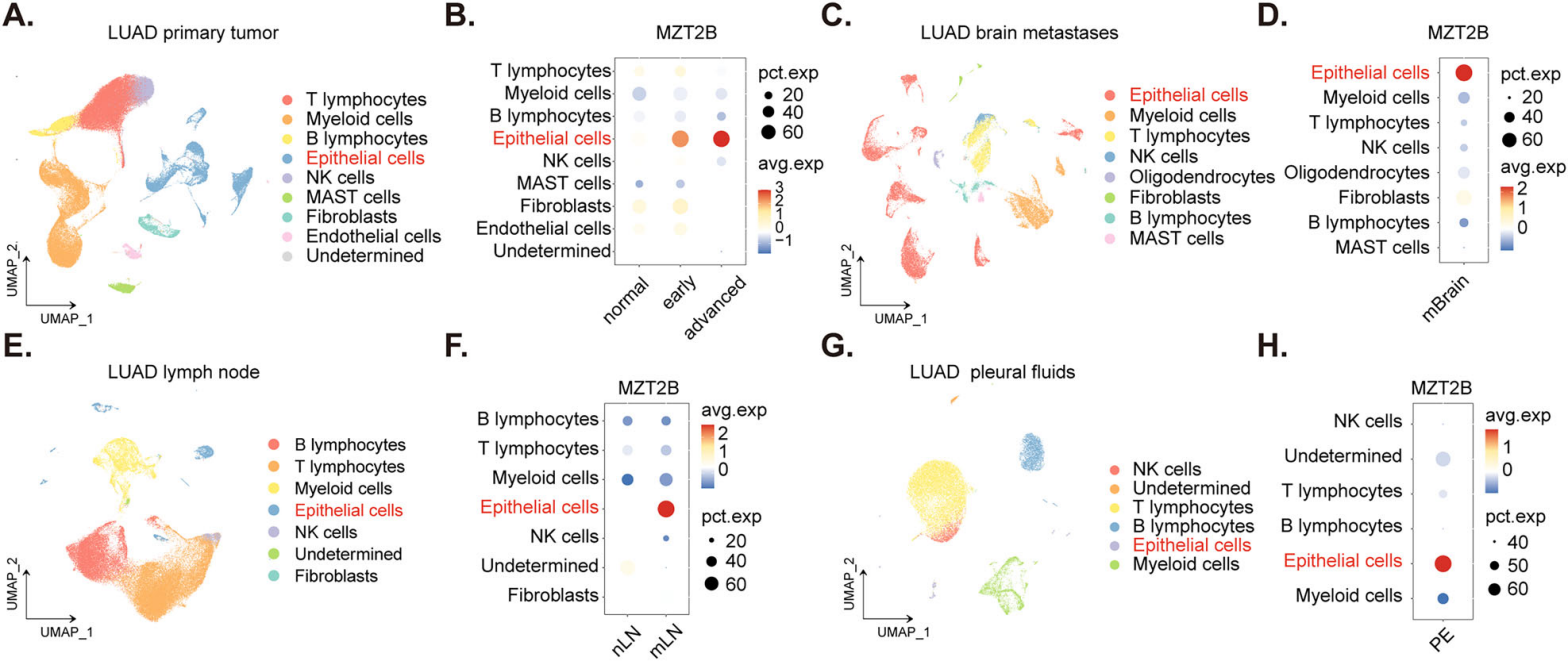
*MZT2B* is consistently overexpressed in malignant epithelial cells across primary and metastatic sites in lung adenocarcinoma (LUAD). UMAP visualization of primary LUAD tumor single-cell data, colored by cell type annotations (**A**). Dot plot depicting the percentage of cells expressing *MZT2B* and average expression levels across different cell types in primary LUAD tumors, highlighting high *MZT2B* expression in malignant epithelial cells (**B**). UMAP visualization of LUAD brain metastases single-cell data, colored by cell type annotations (**C**). Dot plot showing *MZT2B* expression in different cell types within LUAD brain metastases (**D**). UMAP visualization of LUAD lymph node single-cell data, colored by cell type annotations (**E**). Dot plot illustrating *MZT2B* expression in different cell types within LUAD lymph node samples, with notable enrichment in malignant epithelial cells of metastatic lymph nodes (mLN) compared to non-metastatic lymph nodes (nLN) (**F**). UMAP visualization of LUAD pleural fluid single-cell data, colored by cell type annotations (**G**). Dot plot highlighting *MZT2B* expression in different cell types within LUAD pleural (PE) fluid samples (**H**).

The expression profile of *MZT2B* within diverse cellular compartments and across various metastatic loci was investigated using single-cell RNA sequencing data from a LUAD patient cohort (GSE131907). Cellular identities were assigned based on annotations previously established by the original authors [23]. Within the primary LUAD tumor microenvironment, as delineated in the single-cell map (Fig. 3A), significant upregulation of *MZT2B* expression was observed within malignant epithelial cells, with expression levels increasing concurrently with disease progression and exhibiting elevated expression in both early and advanced disease stages compared to normal tissue (Fig. 3B). Conversely, *MZT2B* expression remained minimal in the epithelial cells of normal lung tissues (Fig. 3A, B), underscoring its tumor-specific enrichment. Analysis of LUAD brain metastatic lesions (mBrain) similarly demonstrated a pronounced upregulation of *MZT2B* within the malignant epithelial cell clusters (Fig. 3C, D), consistent with its elevated expression in primary tumor epithelial cells. In LUAD lymph node samples, *MZT2B* also exhibited high expression within the epithelial cell populations (Fig. 3E, F). Notably, this elevated expression was particularly prominent in metastatic lymph nodes (mLN) compared to non-metastatic lymph nodes (nLN) (Fig. 3E, F), suggesting a potential role in metastatic progression. Further examination of LUAD pleural fluid samples revealed a consistent pattern, with *MZT2B* demonstrating high expression within the epithelial cell clusters (Fig. 3G, H). Collectively, these findings from various LUAD clinical samples consistently highlight the enriched expression of *MZT2B* within the malignant epithelial cell populations across primary tumors and multiple metastatic sites.

### Elevated MZT2B expression is observed in locally treated human NSCLC tissues and various NSCLC cell types

**Fig. 4:**
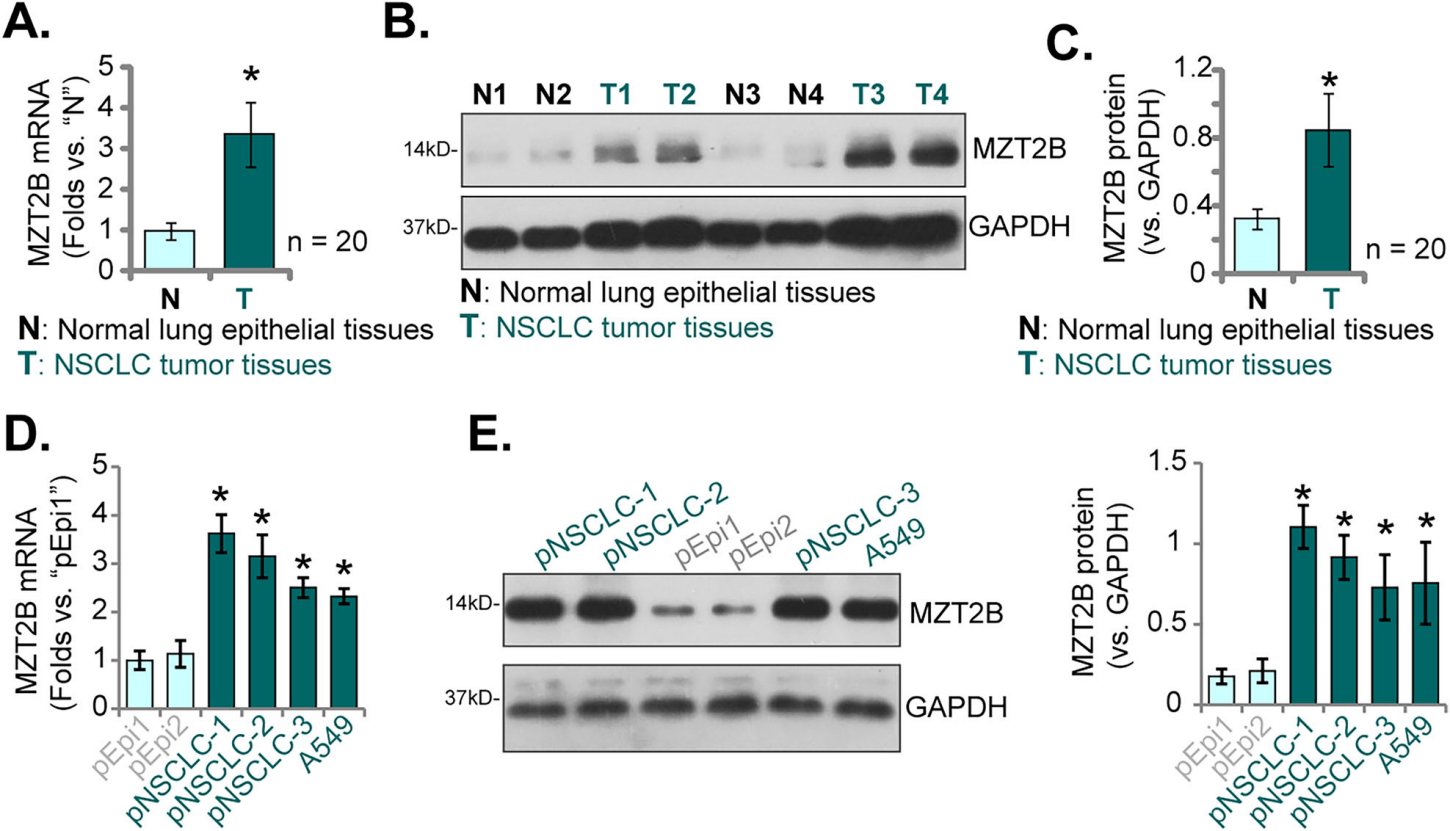
Elevated MZT2B expression is observed in locally-treated human NSCLC tissues and various NSCLC cell types. Quantitative reverse transcription-polymerase chain reaction (qRT-PCR) was performed to assess *MZT2B* mRNA transcript levels in paired NSCLC tumor specimens (T) and adjacent non-malignant lung tissues (N) (*n* = 20, **A**). Representative western blotting analyses were conducted on MZT2B protein lysates from four individual patient pairs to investigate MZT2B protein expression in NSCLC tumor specimens relative to matched normal tissues (**B**). Quantitative densitometric analysis of Western blotting was performed across the entire patient cohort (*n* = 20) to confirm MZT2B protein abundance in NSCLC tumor tissues compared to adjacent non-malignant controls (**C**). qRT-PCR analysis was utilized to measure *MZT2B* mRNA transcript levels in various primary human NSCLC cells (pNSCLC1, pNSCLC2, pNSCLC3) and the A549 cell line, in comparison to normal primary human lung epithelial cells (pEpi1, pEpi2) (**D**). Western blot analysis was subsequently carried out to examine MZT2B protein expression in the NSCLC cells relative to normal lung epithelial controls (**E**). Error bars represent the mean ± standard deviation (SD), with statistical significance *P < 0.05 when comparing to “N” tissues or “pEpi1” cells.

Our investigation was subsequently expanded to examine the differential expression of MZT2B in human NSCLC clinical specimens and relevant cellular models. A cohort of 20 paired fresh NSCLC tumor specimens (“T”) and their adjacent non-malignant lung tissues (“N”), surgically obtained from locally-treated patients, underwent rigorous analysis. qRT-PCR assays unequivocally demonstrated a significant upregulation of *MZT2B* mRNA transcript levels within the malignant tumor tissues when compared to their matched normal counterparts (Fig. 4A). Complementary Western blot analyses, exemplified by four individual patient pairs, consistently revealed higher MZT2B protein expression in the tumor specimens, providing initial evidence of concordant protein upregulation (Fig. 4B). Quantitative assessment of immunoblots across the entire patient cohort (*n* = 20) further corroborated a significant elevation of MZT2B protein abundance in NSCLC tumor tissues relative to the adjacent non-malignant controls (Fig. 4C). To further corroborate these findings, MZT2B expression profiles were evaluated in established in vitro NSCLC cell models. Comparative analysis of multiple primary human NSCLC cells (pNSCLC1, pNSCLC2, pNSCLC3, derived from three distinct informed consent patients [26, 27]) and the A549 cell line, against normal primary human lung epithelial cells (pEpi1, pEpi2 [26, 27]), indicated significantly higher *MZT2B* mRNA transcript levels across all examined NSCLC cell types (Fig. 4D). Consistently, Western blotting analysis further confirmed a markedly elevated MZT2B protein expression in the panel of NSCLC cells relative to their normal lung epithelial controls (Fig. 4E). These results deliver unequivocal and compelling evidence that MZT2B expression is markedly and persistently elevated at both the mRNA and protein levels in human NSCLC clinical specimens and representative cell models, demonstrating a consistent pattern when compared to their non-malignant counterparts.

### MZT2B silencing impedes viability, proliferation, migration, and induces cell cycle arrest in primary and immortalized NSCLC cells

**Fig. 5:**
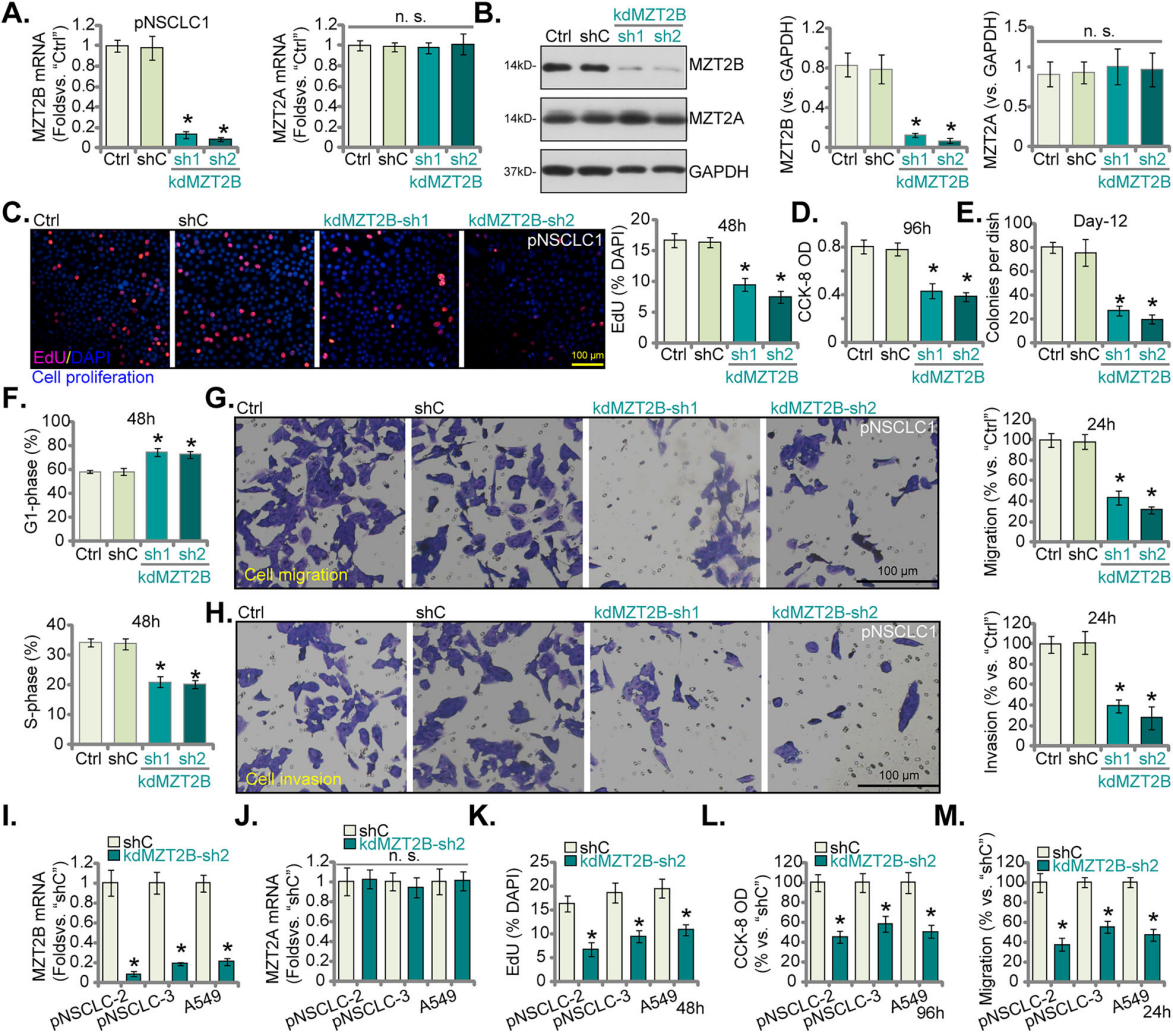
MZT2B silencing impedes viability, proliferation, migration, and induces cell cycle arrest in primary and immortalized NSCLC cells. Primary human non-small cell lung cancer (pNSCLC1) cells were engineered to stably express lentivirus-delivered shRNAs targeting MZT2B (“kdMZT2B-sh1” or “kdMZT2B-sh2”, nonoverlapping sequences) or a scrambled non-sense control shRNA (“shC”). Relative mRNA levels of *MZT2B* and *MZT2A* were determined (**A**). MZT2B and MZT2A protein expression was assessed by immunoblot analysis (**B**). Following a cultivation period, the nuclear EdU incorporation, indicative of DNA synthesis and cell proliferation, was quantified (**C**). Cellular viability was evaluated via CCK-8 assay optical density measurements (**D**). Colony formation was measured using clonogenic assays (**E**). Cell cycle distribution was analyzed by flow cytometry (**F**). Cell migration (**G**) and invasion (**H**) were quantified using Transwell chambers. The analogous experiments were conducted in additional primary human NSCLC cells (pNSCLC2 and pNSCLC3), as well as the immortalized A549 cell line, engineered to maintain stable expression of lentivirus-delivered kdMZT2B-sh2 or shC. The relative mRNA levels of *MZT2B* (**I**) and *MZT2A* (**J**) were determined. The nuclear EdU incorporation (**K**) and CCK-8 optical density measurements (**L**) were quantified. Cell migration (**M**) was assessed via Transwell chambers. “Ctrl” denotes parental control cells. Error bars represent the mean ± standard deviation (SD), with statistical significance (*P < 0.05 when comparing to “shC” cells). “n.s.” stands for *P* > 0.05. All experiments presented in this figure were independently replicated five times (*n* = 5, biological repeats) and consistently yielded similar results. Scale bar = 100 μm.

To elucidate the functional implications of MZT2B in NSCLC, we initiated our investigation by scrutinizing the impact of MZT2B transcriptional attenuation within the primary NSCLC cells (pNSCLC1, [17–21]). As depicted in Fig. 5A, genetic silencing of *MZT2B*, achieved through two independent shRNA constructs (kdMZT2B-sh1 and kdMZT2B-sh2), resulted in a profound diminution in *MZT2B* mRNA levels relative to both parental control (Ctrl) and scrambled shRNA control (shC) pNSCLC1 cells. Concomitantly, the expression of *MZT2A* mRNA remained largely unaltered subsequent to MZT2B knockdown. *MZT2A* was included as a control to validate the specificity of our experimental tools, confirming that the genetic manipulations were exclusively targeting MZT2B without affecting its related paralog in the same gene family. Immunoblot analysis corroborated a substantial reduction in MZT2B protein abundance in kdMZT2B-sh1 and kdMZT2B-sh2 pNSCLC1 cells (Fig. 5B), whereas MZT2A protein levels evinced no statistically significant perturbation. We then assessed the impact of MZT2B knockdown on cell proliferation in pNSCLC1 cells. Figure 5C illustrated that MZT2B knockdown (kdMZT2B-sh1 and kdMZT2B-sh2) led to a significant reduction in nuclear EdU incorporation, indicating decreased DNA synthesis and proliferation. Similarly, CCK-8 cell viability optical values were significantly reduced in kdMZT2B-sh1 and kdMZT2B-sh2 pNSCLC1 cells (Fig. 5D). Furthermore, clonogenic assays revealed a significant decrease in colony formation in MZT2B knockdown pNSCLC1 cells by day 12 (Fig. 5E). Cell cycle profiling, as presented in Fig. 5F, revealed that MZT2B knockdown precipitated an augmented representation of pNSCLC1 cells within the G1 phase, coupled with a corresponding reduction in the S phase populace, thereby causing a G1-S phase cell cycle arrest (Fig. 5F). The migratory and invasive capabilities of pNSCLC1 cells were also studied. As shown in Fig. 5G, MZT2B knockdown significantly inhibited cell migration. Similarly, Matrigel Transwell invasion assays revealed a significant reduction in cell invasion in kdMZT2B-sh1 and kdMZT2B-sh2 pNSCLC1 cells (Fig. 5H).

To further substantiate these compelling observations, analogous experimental paradigms were replicated in two additional primary NSCLC cells (pNSCLC2 and pNSCLC3), as well as the established A549 cell line. Consonant with our findings in pNSCLC1 cells, *MZT2B* mRNA levels were demonstrably suppressed by kdMZT2B-sh2 across all three cells (pNSCLC2, pNSCLC3, and A549) (Fig. 5I), while *MZT2A* mRNA levels persisted without appreciable change (Fig. 5J). Moreover, MZT2B silencing in pNSCLC2, pNSCLC3, and A549 cells culminated in a significant reduction of EdU-positive nuclei ratio (Fig. 5K). Furthermore, CCK-8 assays underscored a pronounced attenuation in cell viability in MZT2B knockdown pNSCLC2, pNSCLC3, and A549 cells (Fig. 5L). Finally, mirroring our earlier findings in pNSCLC1 cells, MZT2B knockdown also exerted a potent inhibitory effect on cellular migration in these NSCLC cells (Fig. 5M). Therefore, our results showed that silencing of MZT2B results in decreased cell viability, impaired proliferative capacity, cell cycle arrest, and reduced migratory potential in both primary and immortalized NSCLC cells.

### MZT2B silencing elicits robust apoptotic cell death in NSCLC cells

**Fig. 6:**
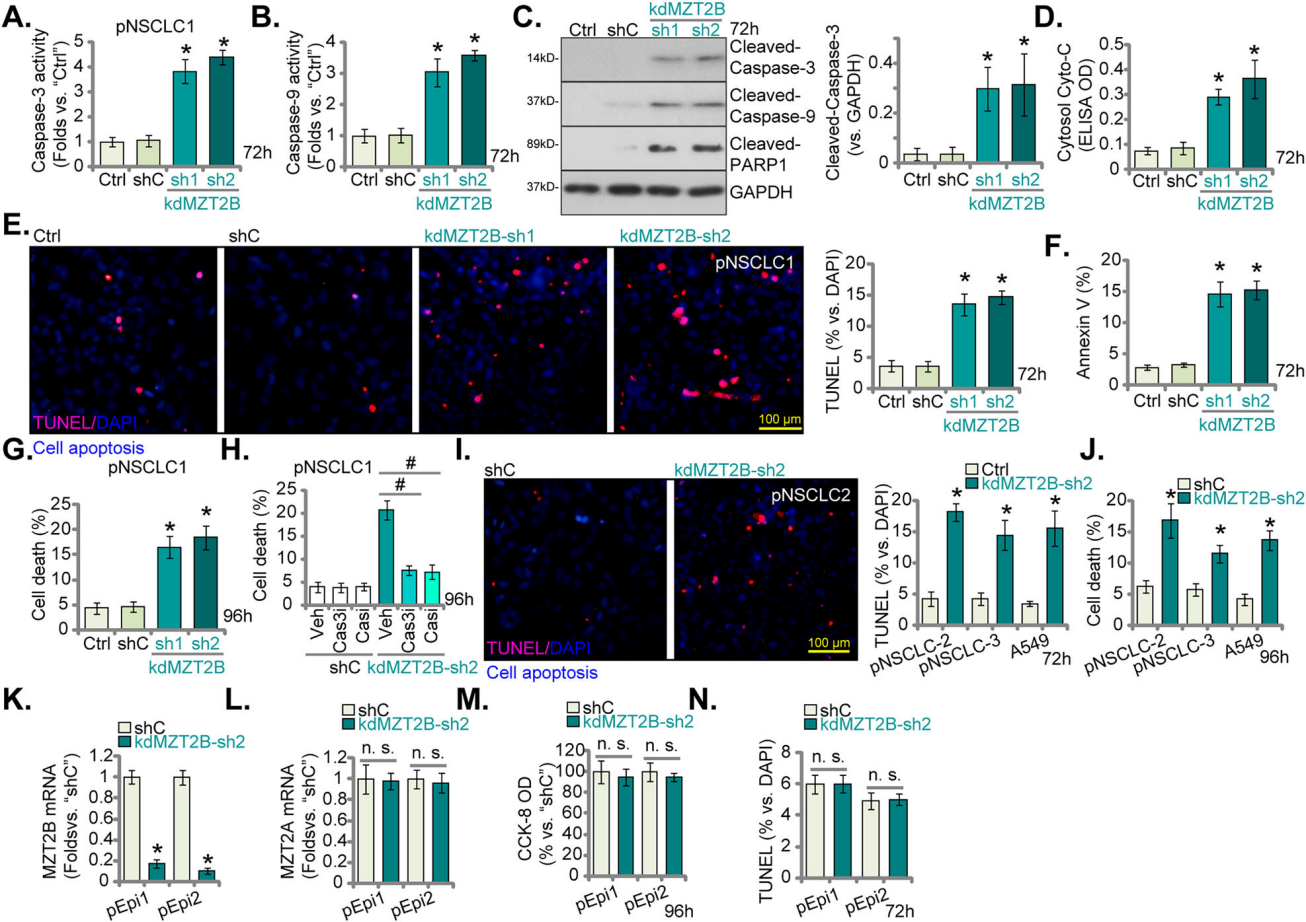
MZT2B silencing elicits robust apoptotic cell death in NSCLC cells. Primary human non-small cell lung cancer (pNSCLC1) cells were engineered to stably express lentivirus-delivered shRNAs targeting MZT2B (“kdMZT2B-sh1” or “kdMZT2B-sh2”, nonoverlapping sequences) or a scrambled non-sense control shRNA (“shC”). Following a cultivation period, Caspase-3 activity (**A**) and Caspase-9 activity (**B**) were assessed. The expression of apoptosis-associated proteins (cleaved Caspase-3, cleaved Caspase-9, and cleaved PARP1) was detected b (**C**). Cytosolic cytochrome c release was measured (**D**). Cellular apoptosis was examined and quantified via the nuclear TUNEL staining (**E**) and Annexin V/Propidium Iodide (PI) FACS (**F**) assays. Overall, cell death was determined by Trypan blue exclusion assay, with results quantified (**G**). The effect of a Caspase-3 specific inhibitor (z-DEVD-fmk, Cas3i, 400 μM) or a pan-caspase inhibitor (z-VAD-fmk, Casi, 400 μM) on cell death in kdMZT2B-sh2-expressing pNSCLC1 cells or shC cells was investigated (**H**). Similar experiments were conducted in primary NSCLC cells (pNSCLC2 and pNSCLC3) and the established A549 cell line, following MZT2B knockdown by kdMZT2B-sh2. The nuclear TUNEL staining (**I**), and overall cell death (**J**) were similarly assessed. Primary human lung epithelial cells (pEpi1 and pEpi2) were engineered to stably express lentivirus-delivered kdMZT2B-sh2 or shC. Relative mRNA levels of *MZT2B* (**K**) and *MZT2A* (**L**) were determined. Cell viability was assessed by CCK-8 assay (**M**). Nuclear TUNEL staining of cell apoptosis assay was carried out, with results quantified (**N**). “Ctrl” denotes parental control cells. Error bars represent the mean ± standard deviation (SD), with statistical significance (*P < 0.05 when comparing to “shC” cells). ^#^*P* < 0.05 (**H**). “n.s.” stands for *P* > 0.05. All experiments presented in this figure were independently replicated five times (*n* = 5, biological repeats) and consistently yielded similar results. Scale bar = 100 μm.

To further delineate the cellular consequences of MZT2B depletion, we investigated its impact on apoptotic pathways in pNSCLC1 cells. MZT2B knockdown (kdMZT2B-sh1 and kdMZT2B-sh2, see Fig. 4) elicited a significant increase in caspase-3 activity and caspase-9 activity in pNSCLC1 cells (Fig. 6A, B), indicating activation of the intrinsic apoptotic pathway. Western blot analysis further corroborated these findings, revealing elevated levels of cleaved Caspase-3, cleaved Caspase-9, and cleaved PARP1 [poly(ADP-ribose) polymerase 1] in MZT2B-silencing pNSCLC1 cells (Fig. 6C). We also observed a significant release of cytochrome c into the cytoplasm in MZT2B knockdown pNSCLC1 cells (Fig. 6D). Direct assessment of apoptosis via TUNEL staining demonstrated a substantial increase in TUNEL-positive pNSCLC1 cells following MZT2B knockdown (Fig. 6E). Flow cytometric analysis using Annexin V/Propidium Iodide (PI) staining confirmed a moderate but significant increase in Annexin V-positive (apoptotic) cell populations in MZT2B-silenced pNSCLC1 cells at 72 h (Fig. 6F). Furthermore, an increased overall cell death, tested via Trypan blue staining assay, was observed at 96 h upon MZT2B knockdown (Fig. 6G). Importantly, treatment with a caspae-3 specific inhibitor (zDEVD-fmk) or a pan-caspase inhibitor (z-VAD-fmk) significantly attenuated the increase in cell death induced by MZT2B knockdown (Fig. 6H), confirming the caspase-dependent nature of this phenomenon.

To ascertain the generalizability of these apoptotic effects, similar experiments were conducted in the primary NSCLC cells (pNSCLC2 and pNSCLC3) and the established A549 cell line. Consistent with the findings in pNSCLC1 cells, MZT2B knockdown by kdMZT2B-sh2 (see Fig. 5) in pNSCLC2/3 and A549 cells also led to an increase in apoptotic cells following MZT2B silencing (Fig. 6I), and a significant increase in overall cell death (Fig. 6J). Conversely, to evaluate the effect of MZT2B silencing in non-cancerous lung epithelial cells, we utilized the pEpi1 and pEpi2 primary human lung epithelial cells. Silencing of *MZT2B* by kdMZT2B-sh2 (Fig. 6K) in these non-cancerous cells did not significantly alter *MZT2A* mRNA levels (Fig. 6L), nor did it impact cell viability as assessed by CCK-8 assay (Fig. 6M), or significantly increase apoptotic (TUNEL-positive) cells (Fig. 6N). These collective findings unequivocally demonstrate that MZT2B silencing elicits robust apoptotic cell death in NSCLC cells through the activation of the intrinsic caspase cascade, without exhibiting similar effects in non-cancerous lung epithelial cells.

### CRISPR/Cas9-mediated knockout of MZT2B exerts potent anti-cancer activity in primary NSCLC cells

**Fig. 7:**
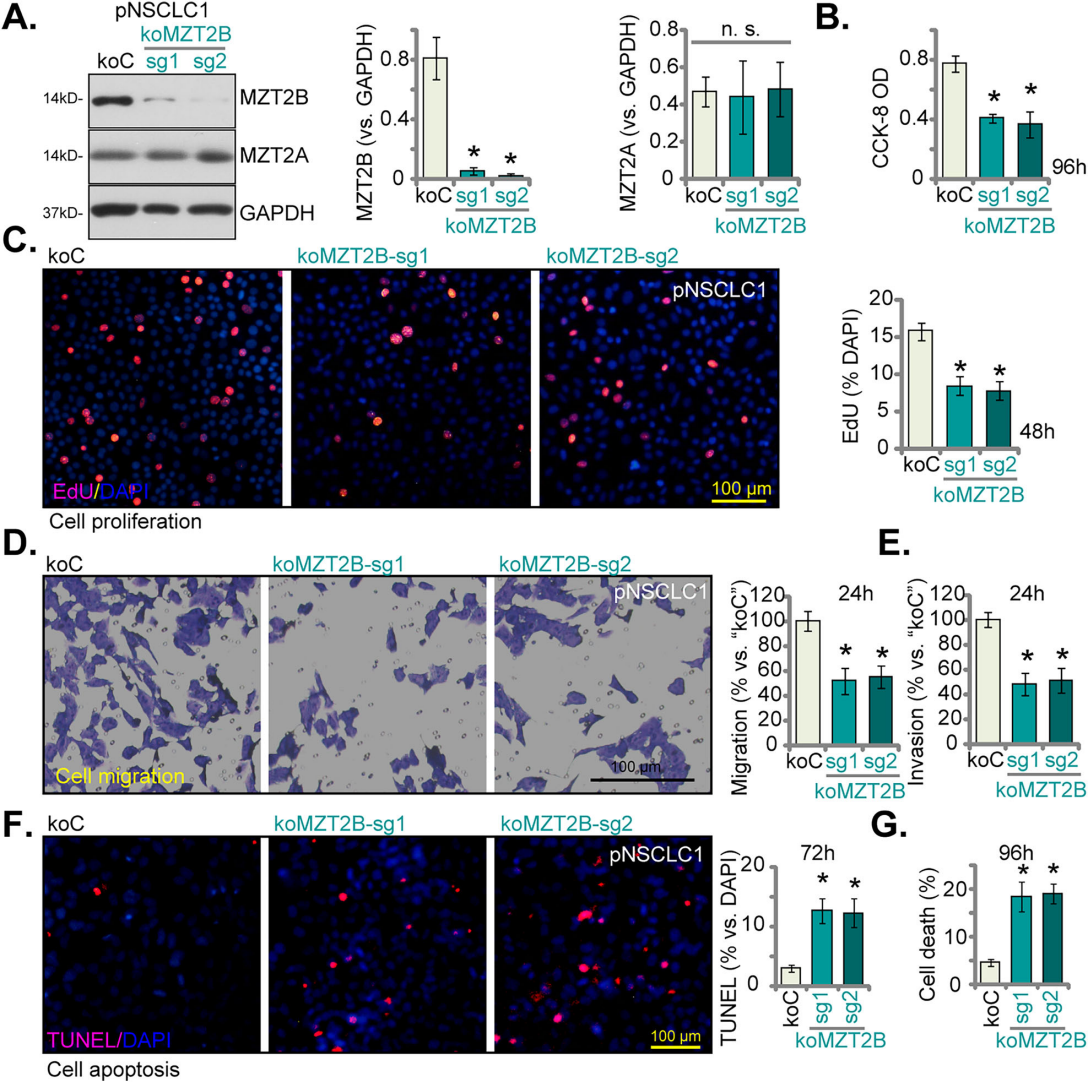
CRISPR/Cas9-mediated knockout of MZT2B exerts potent anti-cancer activity in primary NSCLC cells. Primary human non-small cell lung cancer (pNSCLC1) cells were engineered to stably express a lentivirus-packed Cas9 construct along with the CRISPR/Cas9-MZT2B-KO constructs with two different sgRNAs, “koMZT2B-sg1” or “koMZT2B-sg2”, while control cells expressed a lentivirus-packed Cas9 construct with a CRISPR/Cas9-control sgRNA construct (“koC”). MZT2B and MZT2A protein expression was determined by Western blot analysis (**A**). Following cultivation for indicated time periods, cell viability was assessed via CCK-8 assay (**B**). Nuclear EdU incorporation, indicative of proliferative capacity, was quantified (**C**). Cell migration (**D**) and invasion (**E**) were measured using Transwell chambers. Cellular apoptosis was examined by quantifying nuclear TUNEL staining ratio (**F**). Overall cell death was determined by Trypan blue exclusion assay, with results quantified (**G**). Error bars represent the mean ± standard deviation (SD), with statistical significance (*P < 0.05 when comparing to “sgC” cells). “n.s.” stands for *P* > 0.05. All experiments presented in this figure were independently replicated five times (*n* = 5, biological repeats) and consistently yielded similar results. Scale bar = 100 μm.

To definitively ascertain the indispensable role of MZT2B in NSCLC progression, we employed CRISPR/Cas9 gene editing technology to achieve a complete knockout of MZT2B in primary pNSCLC1 cells. As demonstrated in Fig. 7A, via a lentiviral CRISPR/Cas9 gene knockout construct, two distinct single guide RNAs (koMZT2B-sg1 and koMZT2B-sg2) effectively abrogated MZT2B protein expression, as confirmed by Western blot analysis, without discernibly affecting MZT2A protein levels (Fig. 7A). The functional consequences of MZT2B gene ablation were then rigorously investigated. MZT2B knockout led to a significant attenuation of cell viability as measured by CCK-8 assay (Fig. 7B). Furthermore, cell proliferative capacity, assessed by the nuclear EdU incorporation, was profoundly inhibited in MZT2B knockout pNSCLC1 cells (Fig. 7C), demonstrating an anti-proliferative effect. The migratory and invasive potential of pNSCLC1 cells was also significantly compromised upon MZT2B knockout. Transwell migration assays revealed a marked reduction in cellular migration at 24 h in both koMZT2B-sg1 and koMZT2B-sg2 primary cells (Fig. 7D). Similarly, Matrigel Transwell invasion assays underscored a substantial decrease in the invasive capabilities of MZT2B-KO pNSCLC1 cells (Fig. 7E). Consistent with our earlier observations from MZT2B silencing, direct assessment of cell apoptosis via TUNEL staining revealed an increase in TUNEL-positive cells in MZT2B knockout pNSCLC1 primary cells (Fig. 7F). These pro-apoptotic events culminated in an increase in overall cell death at 96 h following MZT2B knockout (Fig. 7G). These findings, employing a complementary genetic knockout approach, unequivocally corroborate that MZT2B is a critical driver of NSCLC cell proliferation, migration, and invasion, and its elimination induces apoptotic cell death in primary NSCLC cells.

### MZT2B overexpression actively promotes the aggressive phenotypes of NSCLC cells

**Fig. 8:**
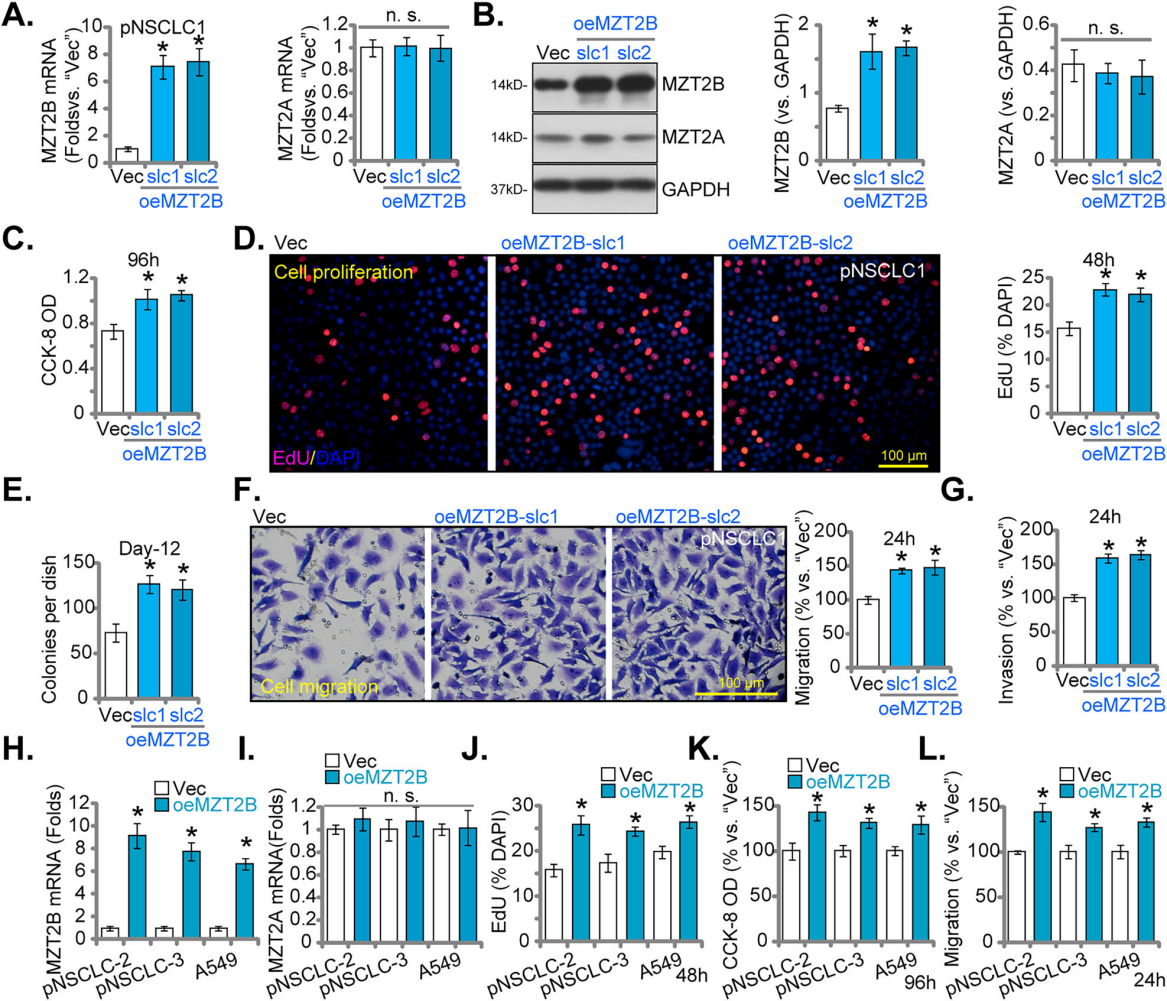
MZT2B overexpression actively promotes aggressive phenotypes of NSCLC cells. Primary human non-small cell lung cancer (pNSCLC1) cells were transfected with a lentiviral MZT2B overexpressing construct to establish stable cell populations (“oeMZT2B-slc1” and “oeMZT2B-slc2”, two selection) or a control vector (“Vec”). Relative levels of *MZT2B* and *MZT2A* mRNA and proteins were determined (**A**, **B**). Following cultivation for indicated time periods, cell viability was examined via CCK-8 assay (**C**). Nuclear EdU incorporation, indicative of DNA synthesis and cell proliferation, was quantified (**D**), with colony formation assessed using clonogenic assays, and results quantified (**E**); Cell migration (**F**) and invasion (**G**) were quantified via Transwell chambers. Additional primary human NSCLC cells (pNSCLC2, pNSCLC3) and the immortalized A549 cell line were also transfected with a lentiviral MZT2B overexpressing construct (“oeMZT2B”) or a control vector (“Vec”). Relative mRNA levels of *MZT2B* (**H**) and *MZT2A* (**I**) were determined. Cell proliferation, tested via nuclear EdU incorporation (**J**) and cell viability, tested via CCK-8 assay (**K**), were quantified. Cell migration (**L**) was assessed via Transwell chambers. “Vec” denotes vector control cells. Error bars represent the mean ± standard deviation (SD), with statistical significance (*P < 0.05 when comparing to “Vec cells). “n.s.” stands for *P* > 0.05. All experiments presented in this figure were independently replicated five times (*n* = 5, biological repeats) and consistently yielded similar results. Scale bar = 100 μm.

To further validate the oncogenic role of MZT2B, we investigated the consequences of its overexpression in NSCLC cells. We established two stable cell populations, termed oeMZT2B-slc1 and oeMZT2B-slc2, by transfecting pNSCLC1 cells with a lentiviral MZT2B overexpressing construct and subsequently performing stable cell selection. As depicted in Fig. 8A, both oeMZT2B-slc1 and oeMZT2B-slc2 cells exhibited significantly elevated *MZT2B* mRNA levels compared to control (Vec) cells, while *MZT2A* mRNA levels was unchanged (Fig. 8A). Western blot analysis confirmed a marked increase in MZT2B protein abundance in these overexpressing pNSCLC1 cells, with no significant alteration in MZT2A protein levels (Fig. 8B). MZT2B overexpression significantly augmented cellular proliferation. CCK-8 assays demonstrated a substantial increase in cell viability in oeMZT2B-slc1 and oeMZT2B-slc2 pNSCLC1 cells (Fig. 8C). Similarly, EdU incorporation was significantly enhanced, indicating increased DNA synthesis and augmented cell proliferation (Fig. 8D). Furthermore, clonogenic assays showed a significant increase in the colony-forming capacity of after MZT2B overexpression (Fig. 8E). The migratory and invasive potentials of pNSCLC1 cells were also significantly enhanced by MZT2B overexpression (Fig. 8F, G).

To broaden the generalizability of these findings, the effects of MZT2B overexpression were also examined in additional primary NSCLC cells (pNSCLC2, pNSCLC3) and the established A549 cell line. Similar to pNSCLC1 cells, *MZT2B* mRNA levels were significantly elevated in *MZT2B*-overexpressing pNSCLC2, pNSCLC3, and A549 cells (Fig. 8H), with no appreciable change in *MZT2A* mRNA levels (Fig. 8I). Importantly, MZT2B overexpression in these additional cell types consistently led to a significant increase in nuclear EdU incorporation at (Fig. 8J), confirming its pro-proliferative effect. Moreover, CCK-8 assays showed a significant increase in cell viability in oeMZT2B NSCLC cells (Fig. 8K). Transwell assays in these cell types demonstrated a significant enhancement of cellular migration upon MZT2B overexpression (Fig. 8L). Collectively, these results demonstrate that MZT2B overexpression actively promotes the aggressive phenotypes of proliferation, migration, and invasion in NSCLC cells.

### MZT2B is important for mitochondrial respiration and overall function possibly via regulating COX5B expression

**Fig. 9:**
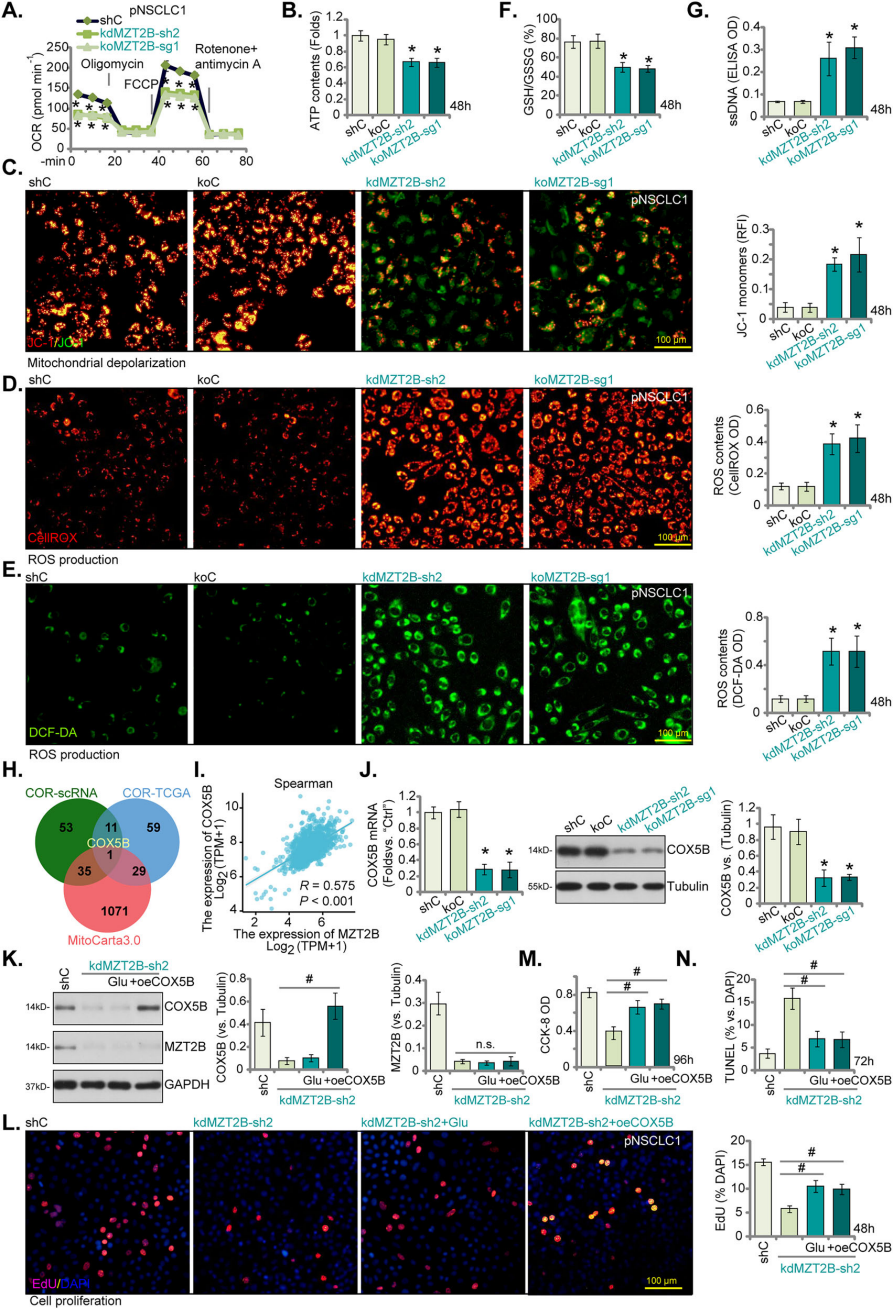
MZT2B is important for mitochondrial respiration and overall function possibly via regulating COX5B expression. Primary human non-small cell lung cancer (pNSCLC1) cells were engineered for MZT2B knockdown (by lentivirus-packed shRNA construct, “kdMZT2B-sh2”) or knockout (by CRISPR/Cas9-MZT2B-KO construct with one sgRNA, “koMZT2B-sg1”), while control cells (“shC” for knockdown and “koC” for knockout) expressed a lentivirus-packed shRNA or Cas9 construct with a control sgRNA, respectively. Following cultivation for indicated time periods: Oxygen consumption rate (OCR) was measured by Seahorse analyzer (**A**). Cellular ATP content was determined (**B**), and mitochondrial depolarization evaluated based on the intensity of JC-1 monomers’ fluorescence (**C**). Reactive oxygen species (ROS) production was measured by CellROX and DCF-DA intensities (**D**, **E**). The glutathione (GSH/GSSG) ratio was quantified (**F**), with single-stranded DNA (ssDNA) intensity measured by ELISA (**G**); Expression of COX5B was also shown (**J**). A Venn diagram illustrating the intersection of genes positively correlated with *MZT2B* from cancer cell subgroup of NSCLC single-cell RNA-seq (COR-scRNA), TCGA bulk RNA-seq (COR-TCGA), and mitochondrial genes (MitoCarta3.0) is shown (**H**); A correlation plot showing the co-expression relationship between *MZT2B* and *COX5B* in TCGA NSCLC datasets is presented (**I**). In experiments where pNSCLC1 cells with MZT2B knockdown (kdMZT2B-sh2) were treated with increased glucose concentration (“Glu”, increasing 10 mM) or had COX5B expression restored by a lentiviral construct (“oeCOX5B”): Western blot analysis showing the protein expression of MZT2B and COX5B is provided (**K**). Cell proliferation was assessed by EdU incorporation (**L**). Cell viability was assessed via CCK-8 assay (**M**), with cell apoptosis tested via nuclear TUNEL staining (**N**) after culture for indicated times. Error bars represent the mean ± standard deviation (SD), with statistical significance (*P < 0.05 when comparing to “shC” cells). ^#^*P* < 0.05 (**K**–**N**). “n.s.” stands for *P* > 0.05. All experiments presented in this figure were independently replicated five times (*n* = 5, biological repeats) and consistently yielded similar results. Scale bar = 100 μm.

Leveraging bioinformatic predictions that linked MZT2B to mitochondrial function and metabolic mechanisms in NSCLC carcinogenesis (Fig. 2), we investigated its functional role in mitochondrial respiration and cellular metabolism. Our comprehensive findings unequivocally demonstrated that both MZT2B knockdown (via kdMZT2B-sh2, Figs. 5, 6) and knockout (via koMZT2B-sg1, Fig. 7) robustly impaired mitochondrial respiration and overall function (Fig. 9A–G). Specifically, MZT2B depletion led to a significant reduction in the oxygen consumption rate (OCR) across various mitochondrial states, including basal and maximal respiration (Fig. 9A). This impairment in respiratory capacity was further corroborated by a concomitant decrease in cellular ATP content (Fig. 9B). Moreover, assessment of mitochondrial membrane potential, using JC-1 staining, revealed a significant reduction upon MZT2B manipulation, indicative of mitochondrial depolarization (Fig. 9C). Consistently, the production of reactive oxygen species (ROS), measured via using CellROX and DCF-DA dyes, was markedly increased in MZT2B-deficient pNSCLC1 cells (Fig. 9D, E), signifying heightened oxidative stress. Further supporting this oxidative imbalance, the glutathione (GSH/GSSG) ratio, a crucial indicator of cellular redox state, was decreased, suggesting altered redox homeostasis (Fig. 9F). An observed increase in single strand DNA (ssDNA) intensity in MZT2B-depleted pNSCLC1 cells provided additional evidence of oxidative damage (Fig. 9G).

To elucidate potential downstream effectors through which MZT2B promotes mitochondrial function in NSCLC cells, we conducted rigorous correlation analyses. Employing an unbiased approach, we first analyzed single-cell RNA sequencing data from cancer cell subpopulations (Fig. 2) to identify the top 100 genes positively correlated with MZT2B expression (termed COR-scRNA). Subsequently, we performed a similar correlation analysis using bulk RNA sequencing data from TCGA NSCLC datasets to identify another set of top 100 positively correlated genes (termed COR-TCGA). The intersection of these two gene sets with a comprehensive list of mitochondrial genes (MitoCarta3.0) revealed a single common candidate: *Cytochrome c oxidase subunit 5B* (*COX5B*) (Fig. 9H). Further analysis of TCGA data confirmed a strong co-expression relationship between MZT2B and COX5B (Fig. 9I). Notably, in pNSCLC1 cells, both MZT2B knockdown (by kdMZT2B-sh2, see Figs. 5 and 6) and knockout (by koMZT2B-sg1, see Fig. 7) significantly decreased the mRNA and protein expression of COX5B (Fig. 9J). This observation strongly suggests that MZT2B plays a pivotal role in regulating expression of COX5B, a key component of mitochondrial complex IV, thereby influencing a critical aspect of mitochondrial electron transport [29, 30].

We then investigated whether modulating COX5B or glucose levels could mitigate the adverse effects of MZT2B depletion. Intriguingly, increasing glucose concentration (Glu) in the cell culture media of kdMZT2B-sh2 pNSCLC1 cells significantly suppressed the anti-cancer effects induced by MZT2B knockdown (Fig. 9K–N). Similarly, the restoration of COX5B expression via a lentiviral construct (“oeCOX5B”) in kdMZT2B-sh2 cells (Fig. 9K) effectively attenuated the impaired cell proliferation (Fig. 9L), decreased cell viability (Fig. 9M), and apoptosis activation (Fig. 9N) observed upon MZT2B knockdown. Collectively, these findings strongly indicate that MZT2B orchestrates mitochondrial function and NSCLC cell progression, at least in part, through its regulation of COX5B and its influence on cellular metabolic processes.

### MZT2B knockdown impairs NSCLC xenograft growth in vivo

**Fig. 10:**
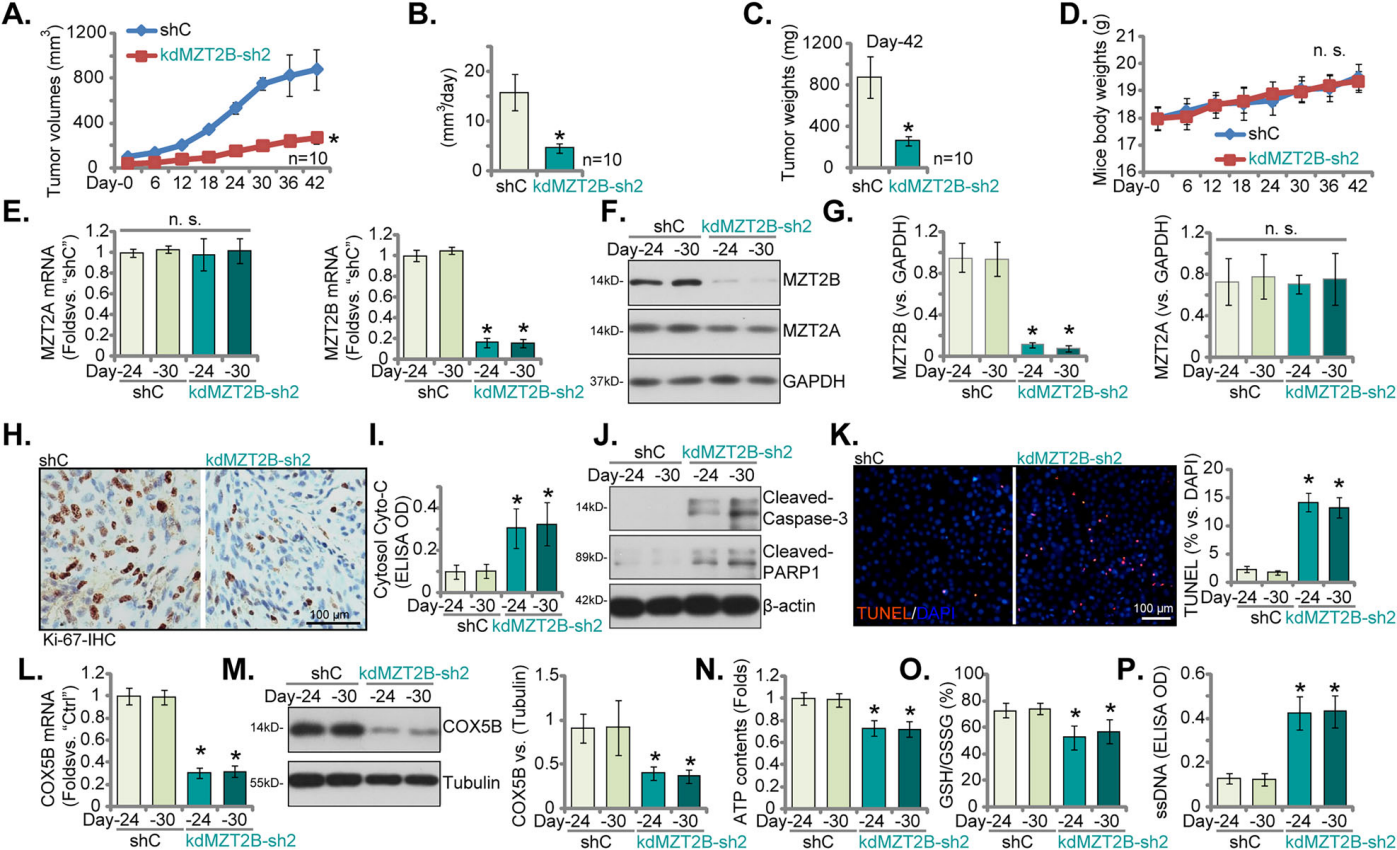
MZT2B knockdown impairs NSCLC xenograft growth in vivo. Analysis of subcutaneous xenografts derived from primary pNSCLC1-1 cells stably expressing control shRNA (shC) or shRNA targeting MZT2B (kdMZT2B-sh2) engrafted into nude mice. Longitudinal monitoring of tumor volume over 42 days post-initiation (Day-0 defined as three weeks post-engraftment) (**A**). Calculation of the derived daily tumor growth (**B**). Assessment of explanted tumor mass at Day-42 (**C**). Longitudinal monitoring of animal body weights (**D**). Validation of target modulation within tumor tissues explanted at Day-24 and Day-30 encompassed qRT-PCR analysis of *MZT2B* and *MZT2A* mRNA levels (**E**) and immunoblotting analysis with quantification of MZT2B and MZT2A protein levels (**F** and **G**). Further analyses of tumor characteristics included IHC assessment of Ki-67-positive proliferating cells in tumor sections (**H**), quantification of cytosolic Cytochrome c levels in tumor lysates (**I**), immunoblotting analysis with quantification of cleaved Caspase-3 and cleaved PARP1 protein levels in tumor lysates (**J**), and TUNEL assay for detection of apoptotic cells in tumor sections (**K**). The *COX5B* mRNA and COX5B protein levels (**L**, **M**) were also analyzed. Analyses of mitochondrial function and redox state performed on lysates from tumors explanted at Day-24 and Day-30 encompassed measurement of total cellular ATP contents (**N**), determination of the cellular GSH/GSSG ratio (**O**), and measurement of ssDNA intensity (**P**). Error bars represent the mean ± standard deviation (SD), with statistical significance (*P < 0.05 when comparing to “shC” group). “n.s.” stands for *P* > 0.05. Data presented in (**A**–**D**) were derived from *n* = 10 mice per group. For (**E**–**P**), analyses were conducted on five distinct tissues randomly selected from each xenograft (*n* = 5). Scale bar = 100 μm.

To evaluate the in vivo significance of MZT2B in NSCLC progression, we established subcutaneous xenograft models in nude mice using pNSCLC1 cells stably expressing either control shRNA (shC) or MZT2B knockdown shRNA (kdMZT2B-sh2, see Figs. 5 and 6). Upon three weeks post-cell inoculation, data recording commenced, designated as “Day-0”. Xenograft tumors derived from kdMZT2B-sh2-expressing pNSCLC1 cells exhibited significantly slower growth compared to those formed by control shRNA-expressing pNSCLC1 cells (Fig. 10A). Throughout the observation period, the kdMZT2B-sh2 group exhibited markedly smaller tumor volumes (Fig. 10A). This resulted in significantly reduced estimated daily tumor growth (Fig. 10B) and lower tumor weights at the study endpoint on Day-42 (Fig. 10C). While the overall mouse body weights remained comparable between the groups (Fig. 10D), indicating no overt systemic toxicity from the MZT2B knockdown. Thus, the substantial reduction in tumor burden highlights the crucial role of MZT2B in NSCLC tumor growth in vivo.

A total of four pNSCLC1 xenografts were analyzed, with one xenograft isolated from each group on both Day-24 and Day-30. Analysis of MZT2B expression within the excised xenograft tumors confirmed effective knockdown at both the mRNA (Fig. 10E) and protein (Fig. 10F) levels in the kdMZT2B-sh2 group compared to controls, validating the experimental model. Expression of MZT2A was however not significant altered (Fig. 10E–G). Furthermore, quantification of Ki-67, a marker of cellular proliferation, showed a significant decrease in proliferative activity within the kdMZT2B-sh2 tumors (Fig. 10H), consistent with their slower growth. Conversely, Cytosolic Cytochrome C levels were elevated in the kdMZT2B-sh2 group xenograft tissues (Fig. 10I), accompanied by increased levels of cleaved Caspase-3 and cleaved PARP1 (Fig. 10J), indicating enhanced apoptosis. The increased nuclear TUNEL staining (Fig. 10K) further confirmed enhanced apoptosis within the kdMZT2B-sh2 xenografts. Mirroring the in vitro findings, MZT2B knockdown in xenograft tumors also led to a significant decrease in *COX5B* mRNA (Fig. 10L) and protein (Fig. 10M) levels, enforcing the in vivo relevance of the MZT2B-COX5B axis. Moreover, the kdMZT2B-sh2 xenografts exhibited reduced ATP content (Fig. 10N), a decreased GSH/GSSG ratio (Fig. 10O), and increased ssDNA intensity (Fig. 10P), collectively indicating compromised mitochondrial function and heightened oxidative stress in vivo. These in vivo results strongly support that MZT2B is a critical regulator of NSCLC tumor growth, largely mediated through its impact on mitochondrial function and cellular metabolism.

## Discussion

NSCLC continues to represent a significant global health burden, characterized by high mortality rates and the persistent challenge of therapeutic resistance [2, 6, 11, 31]. Despite advancements in targeted therapies, a substantial proportion of patients either lack actionable mutations or develop acquired resistance, underscoring the paramount importance of identifying and validating novel molecular targets to improve patient outcomes and overcome current treatment limitations [2, 6, 11, 31]. This study comprehensively characterizes MZT2B as an upregulated pro-cancerous gene in NSCLC, correlating with adverse clinicopathological features and poor prognosis. MZT2B critically drives NSCLC cell proliferation, survival, migration, and tumor growth possibly by maintaining mitochondrial function and respiration, potentially through the regulation of COX5B. It is a promising novel diagnostic/prognostic biomarker and a compelling therapeutic target for NSCLC.

By facilitating the proper formation and function of the mitotic spindle, MZT2B ensures the precise and equal segregation of chromosomes during mitosis [14]. This accurate distribution of genetic material is essential for successful cell division, proliferation, and the maintenance of genomic stability in daughter cells. In this study, we comprehensively establish MZT2B as a novel oncogenic driver and a compelling therapeutic target in NSCLC. Our bioinformatic analyses, leveraging TCGA data, robustly demonstrate that *MZT2B* is significantly upregulated in both LUAD and LUSC tissues. This elevated expression correlates with adverse clinicopathological features, including advanced T and N stages, and is strongly associated with diminished overall survival, highlighting MZT2B’s robust prognostic utility in NSCLC patients. Furthermore, single-cell RNA sequencing analyses unequivocally confirmed MZT2B’s predominant enrichment within malignant epithelial cells, particularly in proliferating carcinoma subsets, and its consistent presence across primary LUAD tumors and various metastatic sites, suggesting its involvement throughout disease progression. In addition, experimental validation in locally-treated human NSCLC clinical specimens and various NSCLC cell types further confirmed consistent MZT2B overexpression.

Our functional investigations rigorously delineate the role of MZT2B in NSCLC malignant phenotypes. In vitro, genetic silencing or CRISPR/Cas9-mediated knockout of MZT2B significantly impeded key malignant behaviors of NSCLC cells, including viability, proliferation, migration, and invasion. This inhibition was accompanied by a clear G1-S phase cell cycle arrest and, importantly, activation of the intrinsic apoptotic pathway. Notably, these detrimental effects were selectively observed in NSCLC cells, with no impact on non-cancerous lung epithelial cells. Conversely, MZT2B overexpression actively promoted aggressive NSCLC phenotypes, further solidifying its oncogenic role. These in vitro findings were powerfully corroborated by in vivo studies, where MZT2B knockdown markedly impaired NSCLC tumor growth in subcutaneous xenograft models, confirming its crucial role in tumor progression. The identification of MZT2B as a consistently upregulated oncogenic driver in NSCLC carries profound clinical implications and emerges as a vulnerability that could be exploited through targeted inhibitors, perhaps in combination with standard NSCLC therapies, including immune checkpoint inhibitors.

Recent research highlights that key mitochondrial proteins are pivotal in NSCLC progression, extending their roles beyond basic energy production to influence metabolic reprogramming, mitochondrial dynamics, and protein quality control [17, 21, 26, 27, 32, 33]. Proteins such as OPA1, involved in mitochondrial fusion, is often amplified in LUAD, promoting tumor growth and immune evasion by maintaining mitochondrial respiratory function [34]. ADCK2 (aarf domain containing kinase 2), a mitochondrial enzyme vital for coenzyme Q biosynthesis and fatty acid metabolism, is consistently overexpressed in NSCLC tissues, linked to reduced overall patient survival [33]. Suppressing ADCK2 profoundly inhibited NSCLC cell viability, proliferation, and motility, disrupted essential mitochondrial functions, and weakened Akt-mTOR signaling, ultimately curbing tumor growth [33]. Similarly, YME1L (YME1 Like 1 ATPase), an inner mitochondrial membrane protease, is upregulated in NSCLC. Silencing or knocking out YME1L curtailed NSCLC cell proliferation and migration, induced apoptosis and mitochondrial dysfunction, and suppressed tumor growth in nude mice [32]. Furthermore, POLRMT (RNA polymerase mitochondrial), an enzyme critical for mitochondrial DNA (mtDNA) transcription and thus essential for mitochondrial protein synthesis and cellular energy production, is overexpressed and demonstrably vital for NSCLC cell growth [21]. Overexpression of MTCH2 (mitochondrial carrier homolog 2) or TIMM23 (translocase of inner mitochondrial membrane 23) similarly promotes NSCLC cell growth by maintaining mitochondrial hyper-function, including enhanced complex I activity and ATP production [17, 26].

Mitochondrial function has emerged as a critical target in NSCLC therapy, moving beyond the traditional view of cancer metabolism to recognize mitochondria’s indispensable roles in tumorigenesis, progression, and drug resistance [35]. Diverse therapeutic strategies are being explored. For instance, mito-Honokiol (Mito-HNK) inhibited respiratory complex I, stimulated ROS production, and suppressed mitochondrial STAT3, effectively suppressing lung cancer development and brain metastases in mouse models [36]. DZ-SIM targets mitochondrial structure and function, proving effective against cisplatin and EGFR TKI-resistant NSCLC xenografts [37]. Mitochondrial complex inhibitors such as metformin and IACS-010759, which target complex I, show preclinical antitumoral effects in NSCLC, though clinical trials have faced challenges with mixed results [38]. Modulating mitochondrial dynamics, particularly inhibiting excessive fission with agents like Mdivi-1, reduced NSCLC cell proliferation and regressed tumor growth in models [39].

A novel finding of this study is the critical involvement of MZT2B in maintaining mitochondrial hyperfunction and cellular respiration in NSCLC cells. Thus, the significance of this study extends beyond NSCLC, illuminating a novel mechanistic axis linking mitotic spindle organization to mitochondrial function in cancer biology. MZT2B silencing or KO led to impaired OCR, reduced ATP production, decreased mitochondrial membrane potential, and disrupted cellular redox homeostasis, evidenced by increased ROS production and an altered glutathione (GSH/GSSG) ratio. Our integrated bioinformatic and experimental approaches identified COX5B as a key downstream effector regulated by MZT2B. COX5B is a nuclear-encoded subunit of Cytochrome c Oxidase (Complex IV), which serves as the terminal enzyme in the mitochondrial electron transport chain [29, 30]. Its fundamental role within the mitochondria is to support oxidative phosphorylation by facilitating the precise transfer of electrons to molecular oxygen [29, 30]. This critical step is intrinsically linked to the pumping of protons across the inner mitochondrial membrane, thereby generating the protonmotive force. This electrochemical gradient subsequently drives ATP synthase to produce the vast majority of cellular ATP, underscoring COX5B’s indispensable contribution to cellular energy production and metabolic equilibrium [29, 30]. The finding that MZT2B regulates COX5B provides a mechanistic link to its role in mitochondrial function and NSCLC progression. We demonstrated that restoring COX5B expression or increasing glucose concentration could significantly attenuate the anti-NSCLC cell effects induced by MZT2B silencing. This suggests that MZT2B promotes NSCLC malignancy, at least in part, by orchestrating mitochondrial function through its regulation of COX5B, thereby supporting the heightened metabolic demands of cancer cells. Thus, by demonstrating that MZT2B promotes aggressive behaviors partially through regulating mitochondrial functions and COX5B upregulation, we provide experimental validation of bioinformatic predictions, filling a gap in understanding how spindle proteins could possibly influence metabolic reprogramming in NSCLC.

Together, MZT2B is consistently upregulated in NSCLC that correlates with adverse clinicopathological features and poor prognosis and critically regulates mitochondrial function and promotes NSCLC progression, in part, by promoting COX5B expression. Prospective clinical studies involving larger NSCLC patient cohorts and diverse ethnic groups are required to further validate MZT2B as a reliable diagnostic, prognostic, or predictive biomarker. Establishing standardized detection methods, optimal cutoff values, and assessing its incremental value over existing biomarkers will be essential for its successful translation into clinical utility. A further comprehensive investigation into the molecular interactions and signaling pathways of MZT2B within NSCLC is also needed. Moreover, future research into small molecule inhibitors or other targeted therapies specifically designed to modulate MZT2B activity in NSCLC is warranted.

## Supplementary information

Figure S1

Original data set.

## Data Availability

All data generated or analyzed during this study are included within the manuscript and its Supplementary Materials.
