# Supplementary figures and images for: MZT2B promotes malignant phenotypes in NSCLC cells by enhancing mitochondrial function and COX5B expression
Source: Cell Death Dis. 2025 Nov 10;16(1):827. doi: 10.1038/s41419-025-08182-y (PMC12603160; doi:10.1038/s41419-025-08182-y)

Figure S1. The uncropped blot images of the study.

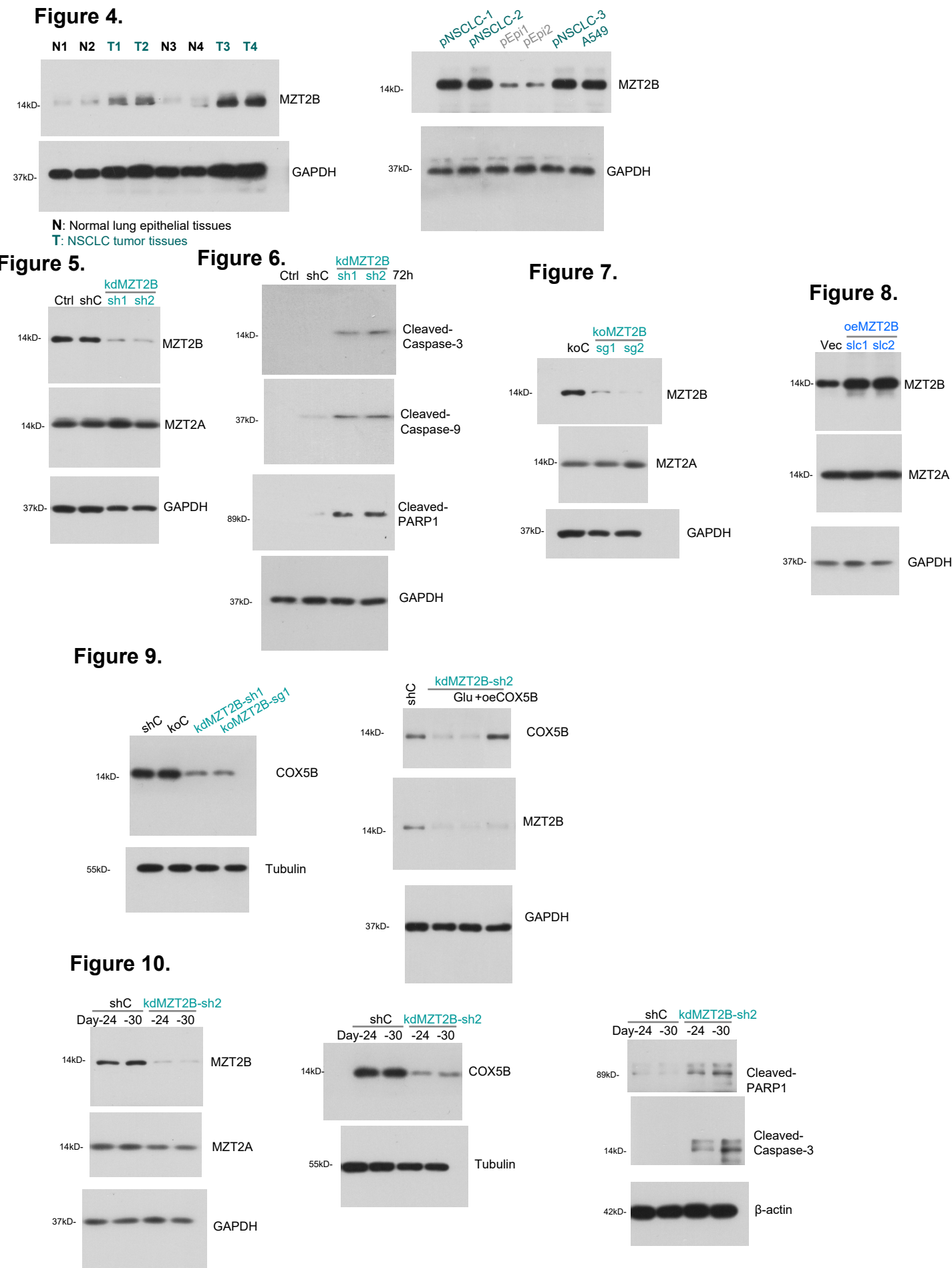

Supplement: Supplementary file 1 — Figure S1 [file 41419_2025_8182_MOESM1_ESM.pdf]
